# Nanoplasmonic Infrared Microarray Sensor Enabling Structural Protein Biomarker‐Based Drug Screening for Neurodegenerative Diseases

**DOI:** 10.1002/advs.202500320

**Published:** 2025-07-28

**Authors:** Deepthy Kavungal, Enzo Morro, Senthil T Kumar, Berkay Dagli, Hilal A Lashuel, Hatice Altug

**Affiliations:** ^1^ Bionanophotonic Systems Laboratory (BIOS) Institute of Bioengineering École Polytechnique Fédérale de Lausanne Lausanne 1015 Switzerland; ^2^ Laboratory of Molecular Neurobiology and Neuroproteomics (LMNN) Institute of Bioengineering École Polytechnique Fédérale de Lausanne Lausanne 1015 Switzerland; ^3^ National Centre for Cell Science Pune 411007 India

**Keywords:** alpha‐synuclein, high‐throughput screening, infrared spectroscopy, metasurfaces, microarray, neurodegenerative diseases, plasmonics

## Abstract

The misfolding of proteins from native monomers into β‐sheet‐rich fibrils via oligomers is a key hallmark of neurodegenerative diseases (NDDs). Identifying and screening drugs that inhibit protein aggregation for early disease intervention remains challenging due to the limitations of existing methods. This work introduces a novel nanoplasmonic infrared microarray sensor for label‐free and high‐throughput drug screening based on structural protein biomarkers in NDDs. The sensor employs 2D arrays of nanoplasmonic units compartmentalized in micropatterned polymeric microwells for high‐throughput protein sensing and secondary structural analysis. The flexibility of the on‐chip integrated microarray sensor is showcased through ultra‐compact 48, 96, and 384 microwell designs, enabling detection from as low as 2 nL of sample volume and with a 100 pg/mL sensitivity in under a minute of in situ measurement. The drug screening capability is validated by assessing multiple drug compounds in a multiplexed manner for their inhibiting effect on aSyn aggregation, an important NDDs protein biomarker. The microarray sensor successfully quantified the secondary structural changes in drug‐treated protein samples, detecting both oligomers and fibrils, which the conventional fluorescence‐based assays failed to do. Thus, the nanoplasmonic microarray sensor is a promising advancement in the NDDs and pharmaceutical research for drug screening.

## Graphical abstract

This work introduces an on‐chip, integrated plasmonic infrared microarray sensor that enables high‐throughput, label‐free drug screening targeting structural protein biomarkers in neurodegenerative diseases. Through ultra‐compact microwell arrays, the sensor achieves ultra‐sensitive, rapid, and multiplexed secondary structural analysis of the effect of drugs on protein aggregation from nanoliter samples, providing detailed structural insights that surpass the capabilities of conventional fluorescence‐based assays.

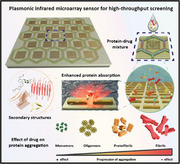

## 1 Introduction

Protein biomarkers have been the subject of intensive studies as targets for diagnostics, treatment monitoring, and drug development due to their critical roles in health and disease.^[^ ^1^, ^2^, ^3^ ^]^ They fold into different structural conformations to acquire their normal function.^[^ ^4^, ^5^ ^]^ Sporadically, proteins can misfold, i.e., the folding process can result in different secondary structures instead of their native form, disrupting their proper function and thereby implicated in various diseases.^[^ ^6^ ^]^ In fact, protein misfolding is an important hallmark of neurodegenerative disorders (NDDs). Despite the heterogeneity of the diseases and different proteins involved in various NDDs including Parkinson's disease (PD, alpha‐synuclein (aSyn)), Alzheimer's disease (AD, amyloid‐beta (Aβ) and tau), amyotrophic lateral sclerosis (ALS, TDP‐43), and prion diseases (prion protein (PrP)), these diseases and structural biomarker proteins share common aggregation mechanisms leading to formation of pathogenic protein with shared structural features.^[^ ^7^, ^8^, ^9^ ^]^ Specifically, the misfolding of monomeric proteins from their native disordered or α‐helical structure into β‐sheet‐enriched fibrillar aggregates occurs via intermediary oligomers, which accumulate in the brain, leading to cellular toxicity, synaptic dysfunction, and ultimately neuronal death.^[^ ^10^ ^]^ The oligomers might exist as a mixture of multiple structural forms, as in the case of PD, where factors such as mutations and the presence of compounds like 4‐hydroxy‐2‐nonenal (HNE), a lipid peroxidation by‐product and Dopamine, a neurotransmitter, influence the relative abundance of each form.^[^ ^11^, ^12^, ^13^ ^]^

Identification of drugs that are effective for the early treatment of NDDs remains challenging to date despite the prevalence of these disorders. An exciting and emerging research direction is to identify drug compounds that can inhibit the aggregation of structural biomarker proteins, thereby halting disease progression at an early stage. Recent research has shown small molecules as an affordable, abundant, and promising class of drug compounds that can hinder the aggregation of NDDs structural protein biomarkers.^[^ ^14^, ^15^ ^]^ Efficient drug screening methods are required that can quantitatively probe the secondary structural state of proteins before and after drug treatment to assess the efficacy and mode of action of the drug compounds, i.e., whether the drugs act at the native, oligomeric or fibrillar states. Since drug development and screening are expensive and time‐consuming, pharmaceutical research favors high‐throughput screening (HTS) methods that are fast, automated and can screen multiple compounds in replicates under different conditions from low sample volumes. Most common HTS platforms meeting such requirements have optical readouts, such as fluorescence, scattering, absorbance, and scintillation and are conventionally implemented in standard multiwell plate setups with configurations of 48, 96, and 384 sample wells.^[^ ^16^ ^]^ Circular dichroism (CD) spectroscopy provides secondary structural information on proteins, and recently, high‐throughput CD platforms have emerged.^[^ ^17^, ^18^ ^]^ However, CD spectroscopy requires a high sample concentration to work in a multiwell plate with volumes of a few hundred microliters and cannot provide accurate quantitative information on secondary structure, especially on β‐sheets. On the other hand, dynamic light Scattering (DLS) can be measured using multiwell plate setups in a label‐free and sensitive manner, providing quantitative information on different protein structural forms based on particle size.^[^ ^19^, ^20^ ^]^ However, DLS is prone to errors due to impurities and salt particles in the solution, requiring additional filtration procedures and complex data analysis.^[^ ^21^, ^22^ ^]^ Alternatively, a fluorescent assay based on Thioflavin T (ThT) – a dye that has preferential binding to amyloid structures containing β‐sheets is favored for studying protein aggregation kinetics, seeding amplification assays, and screening misfolding‐inhibiting drugs.^[^ ^23^, ^24^ ^]^ When ThT is added to a mixture of native proteins and drugs under aggregation‐favorable conditions, a time‐dependent increase in optical readout indicates the formation of β‐sheet‐enriched aggregates as the dye fluoresces upon binding to amyloids.^[^ ^25^, ^26^ ^]^ However, this is a qualitative analysis and cannot provide an absolute measure of β‐sheets content and information on the presence or absence of other secondary structural forms, nor any insight into the relative distribution of oligomeric and fibrillar species in the sample.^[^ ^27^, ^28^ ^]^ Another major limitation of this assay is that many drug compounds may quench the signal of the ThT dye, leading to false positives that can only be identified through additional screening assays. All of these HTS methods (CD, DLS, and ThT) are inherently low‐sensitive, necessitating high sample concentrations (≈mg/ml) and long measurement times. Since these assays are performed in traditional multiwell plates, which are bulky and have large well dimensions, high sample volumes (hundreds of microliters) are also needed.

Infrared (IR) spectroscopy is a powerful optical technique for protein studies as it provides label‐free and non‐destructive chemical‐specific analysis with minimal sample preparation in the mid‐IR fingerprint range of 900–1800 cm^−1^.^[^ ^29^, ^30^ ^]^ In particular, within the distinct protein absorption bands of 1500–1700 cm^−1^, the Amide I band (1600–1700 cm^−1^) encodes quantitative information on different secondary structures such as α‐helix, disordered, β‐sheets, and β‐turns, thereby making it an invaluable tool for protein structural analysis.^[^ ^31^, ^32^ ^]^ Having already been employed to understand and detect protein aggregation in NDDs, IR spectroscopy holds a vast potential yet to be fully leveraged for drug screening based on protein structural biomarkers.^[^ ^33^, ^34^ ^]^ In addition to secondary structure analysis, IR spectroscopy has been extended for HTS using conventional multiwell plates on Fourier transform infrared (FTIR) systems.^[^ ^35^ ^]^ Surface‐enhanced infrared absorption (SEIRA) spectroscopy is an emerging method empowered by nanophotonics to advance the sensing and detection capabilities of IR spectroscopy. SEIRA involves the engineering of optical nanostructures and metasurfaces that can generate high‐intensity and localized electromagnetic fields at their resonant wavelengths. For example, nanostructured substrates made of noble metals utilize the excitation of localized surface plasmon resonances (LSPR) to enhance SEIRA performance.^[^ ^36^, ^37^ ^]^ By tailoring the resonant wavelength of the nanostructure array to overlap with the vibrational bands of the proteins, their absorption fingerprints can be retrieved by many orders of magnitude.^[^ ^38^, ^39^ ^]^ This has enabled the measurement and detection of monolayer proteins for highly sensitive analysis of secondary structure.^[^ ^40^, ^41^ ^]^ Importantly, plasmonic SEIRA provides a unique ability to analyze proteins in their native aqueous environment, endowed by the Plasmon Internal Reflection (PIR) principle, enabling microfluidic integration.^[^ ^42^ ^]^ This has led to real‐time IR measurements and quantitative secondary structural analysis of proteins in situ, including biomarkers for NDDs.^[^ ^43^, ^44^, ^45^ ^]^ Despite recent progress, the adaptation of SEIRA as a high‐throughput optical biosensor for screening and drug development applications is still to be explored.

In this paper, we introduce a novel nanoplasmonic infrared microarray sensor for high‐throughput drug screening that targets structural protein biomarkers in neurodegenerative diseases (NDDs). The sensor features two‐dimensionally patterned metasurface arrays as nanoplasmonic sensing units, each consisting of periodically arranged gold nanorods tailored to provide enhanced detection of proteins and retrieval of the secondary structural information from their distinct Amide I band with high spectral selectivity. The high‐throughput detection feature is realized by precisely micropatterning 2D arrays of polymeric honeycomb‐shaped structures enclosing individual nanoplasmonic sensing units to form microwells. We demonstrate the versatility of our proposed method by fabricating ultra‐compact chip‐based microarrays in 48‐, 96‐, and 384‐microwell designs by precise micro‐nanofabrication techniques. The integrated microarray sensor enables sensitive protein detection from ultra‐small sample volumes down to two nanoliters (nL), which is orders of magnitude smaller than the volumes required in conventional bulky well plates (≈µL). The microarray sensors are highly sensitive, with a detection limit down to 100 pg mL^−1^ and the ability to perform rapid in situ IR measurements lasting less than a minute per microwell. The microarray sensor is validated by evaluating the inhibitory effects of nine clinically relevant drug compounds on aSyn aggregation through structural analysis of drug‐treated protein mixtures, providing quantitative information on the different secondary structural forms present and identifying β‐sheet‐enriched fibrils. The microarray sensor also superiorly identified the formation of distinct morphological, non‐fibrillar aggregates, providing insight into their unique secondary structure composition, where current fluorescence assays fail. Thus, our plasmonic infrared microarray sensor represents a significant advancement in optical detection methods, with broad applications in drug discovery, diagnostics, and biomarker discovery for NDDs.

## 2 Results

### 2.1 Plasmonic SEIRA Microarray Sensor Design and Working Principle

**Figure 1:**
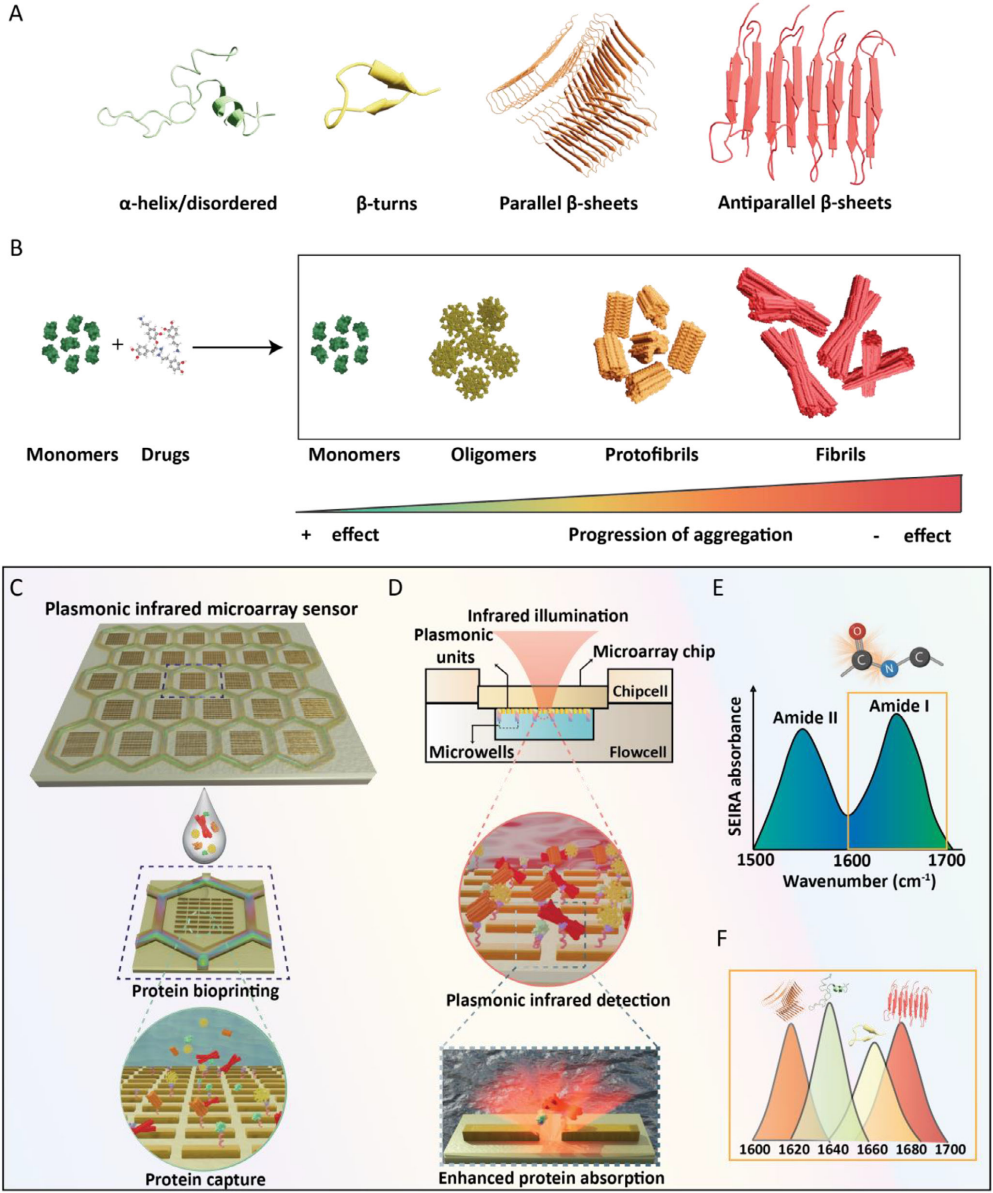
Plasmonic infrared microarray sensor design and working principle. A) Different secondary structure forms of proteins. B) Drug assessment scheme. C) Plasmonic infrared microarray sensor featuring 2D sensing units and microwells of a honeycomb shape. The sensor is spatially bioprinted with the protein drug mixture and captured on the sensor surface. D) Microarray chip is integrated with microfluidic components and illuminated with IR light from the back of the chip, and enhanced absorption is retrieved from the plasmonic units, especially from the nanogaps. E) Amide I and Amide II bands of protein absorption. F) Amide I band envelops distinct absorption signatures of different secondary structure forms.

Proteins often exist in equilibrium or as combinations of different structural motifs, as shown in **Figure** **1**A. Two of the most common secondary structures are the α‐helix and β‐sheets, both of which are formed by hydrogen bonds between the N─H and C═O groups in the peptide backbone.^[^ ^46^ ^]^ In an α‐helix, hydrogen bonding occurs within the same strand, creating a spiral shape, whereas in β‐sheets, it occurs between adjacent strands, forming a pleated structure. If the strands run in opposite directions, they form antiparallel β‐sheets. When no hydrogen bonding occurs between chains, the structure is considered “disordered”. β‐turns connect different secondary structure motifs in the protein.^[^ ^47^ ^]^ Figure 1B shows the drug assessment scheme used in this study. The native monomeric proteins are incubated with different small molecule candidates under aggregation‐favoring conditions (continuous shaking at 37 °C, two days). If the drug has the intended positive effect, the protein mixture remains predominantly in its native form. If the drug has no inhibiting effect, the monomers proceed through different aggregation stages, and the final mixture can contain all the different structural species, such as oligomers, protofibrils, and fibrils, with mixed secondary structure forms or is dominated by one of these forms.

To sense different protein structural species and resolve their mixed secondary structures, we introduce a plasmonic SEIRA microarray sensor, schematically illustrated with its detection principle in Figure 1C–F. The microarray chip contains multiple plasmonic metasurface elements, and these individual sensing units are compartmentalized within SU‐8 honeycomb‐shaped microwells (Figure 1C). The metasurface elements are designed as 2D arrays of plasmonic nanorods combined with nanogaps for sensitive detection of proteins from high‐field enhancement formed between the nanogaps. Each microwell is bioprinted with a few nL of the final product from the drug incubation assay, containing a mixture of different structural forms. The proteins are captured on the sensing elements covalently on the thiol‐functionalized gold nanorods. The microarray chip is integrated with microfluidic components for in situ protein sensing in reflection mode under IR illumination, utilizing the PIR configuration (Figure 1D).^[^ ^42^ ^]^ In this setup, IR light is incident at the backside of the chip, requiring an IR‐transparent substrate such as calcium fluoride (CaF₂), to excite the plasmonic nanoantennas from below. This configuration efficiently couples the incident light to the resonant nanostructures, generating a strong near‐field enhancement at the chip surface with a penetration depth of less than ≈100 nm, which decays exponentially into the aqueous environment in the microfluidics. Therefore, by exciting the nanostructures through the substrate side, the employed PIR‐SEIRA setup significantly reduces the optical path length through water, thereby minimizing background absorption from water while simultaneously enabling sensitive detection of protein structural changes even in aqueous environments.

The resonance excitation facilitates enhanced absorption from the proteins captured on the sensing units, which mainly arises from the proteins close to the nanogaps. The resonance frequency of the metasurface is tuned to overlap with the Amide II (1500–1600 cm^−1^) and Amide I (1600–1700 cm^−1^) absorption bands of the proteins and is retrieved as shown in Figure 1E. In particular, the Amide I band excited through the vibrational modes of C═O and N─H peptide bonds provides insight into the different structural species present in the sample through distinct absorption signatures dictated by individual secondary structures (Figure 1F). For example, parallel β‐sheets have characteristic absorption bands in the lower wavenumbers from 1605–1640 cm^−1^, whereas the α‐helix/disordered forms absorb around 1640–1665 cm^−1^.^[^ ^31^, ^48^ ^]^ While the β‐turns have their signature window from 1665–1680 cm^−1^, the antiparallel β‐sheets have distinct bands in the 1680–1700 cm^−1^ range.^[^ ^49^, ^50^ ^]^ Thus, we rely on the Amide I band for structure‐specific detection and to perform quantitative assessment of secondary structural forms in a high‐throughput manner, which is crucial for drug screening applications in NDDs targeting protein structural biomarkers.

### 2.2 Performance Metrics of the Plasmonic Infrared Microarray Sensors for Biosensing Applications

**Figure 2:**
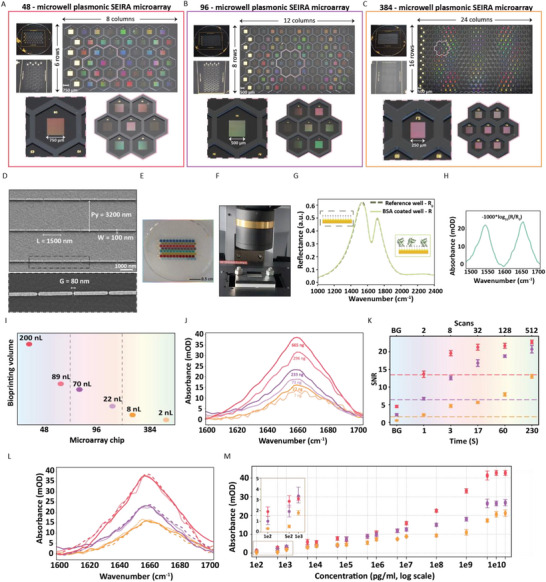
Design and performance metrics of the plasmonic infrared microarray sensors with 48‐, 96‐, and 384‐microwells. Sensor chips are designed with A) 48 microwells, B) 96 microwells, and C) 384 microwells. D) SEM image of the GONGs plasmonic metasurface design. E) Optical image of the 96‐microwell chip spotted with colored buffer and dried. F) Microfluidic integration and FTIR microscopic measurement of the microarray chips. G) Infrared reflectance response of a reference microwell without BSA and the spotted microwell with BSA. H) Amide II and Amide I absorbance bands were retrieved from BSA bound on the microarray sensor surface. I) The minimum and maximum sample volumes required for protein spotting for the different designs. J) The Amide I band absorbance strengths of the different sample volumes used in different designs. K) Calculated SNR for each microarray sensor design for different measurement time/scans. L) Signal variability across three different replicates of the protein microwells within the same microarray chip design. M) Concentration response and Limit of Detection (LoD) ranges of the different microarray designs.

One of the crucial parameters for drug screening applications is the throughput. Commercial multiwell plates used in conventional HTS assays are typically available with 48, 96, and 384 sample wells. Therefore, to be comparable with these throughput levels, we fabricated three different on‐chip integrated microarray sensor chips – each with 48, 96, and 384 microwells, as shown in **Figure** **2**A–C, respectively. The sensor chips are fabricated by two‐step lithography. The first step is the Electron beam (E‐beam) lithography for the nanofabrication of plasmonic sensing units with gold nanorod arrays on an IR transparent CaF_2_ substrate. The second step is photolithography, where the chip with plasmonic units is coated with SU‐8 resist and exposed using a photomask to imprint the honeycomb patterns perfectly enclosing the sensing units and form the microwells (Detailed fabrication process is explained in the Experimental Section). SU‐8 is an excellent choice as it is bio‐compatible and can be easily patterned with a high‐aspect ratio and well‐defined corners, with the ability to withstand subsequent experimental steps and chemical treatments.^[^ ^51^, ^52^ ^]^ Even though the number of microwells differs in each design, the overall sensing surface is limited to a 2 × 2 cm^2^ area of the CaF_2_ substrate with a 3 cm diameter.

The 48‐microwell chip is patterned as 6 × 8 (rows x columns) (Figure 2A), whereas the 96‐microwell chip and 384‐microwell chip are in 8 × 16 (Figure 2B) and 16 × 24 layout (Figure 2C), respectively. We scaled down the size of the microwells and sensing units as we increased the number of sampling wells in a given microarray chip. Each microwell has an approximate diameter of 2, 1.38, and 0.69 mm for the 48‐,96‐, and 384‐microwell chips, respectively and with a common height of 70 µm. The dimensions of the chip and the microwells are orders of magnitude smaller than the traditional multiwell plates, thereby offering a multiplexed on‐chip integrated microarray sensor with an ultra‐compact footprint. The 48‐microwell chip has individual sensing units fabricated as squares of 750 × 750 µm^2^, and the 96‐microwell chip with 500 × 500 µm^2^. The 384‐microwell chip has the smallest sensing unit size of 250 × 250 µm^2^. The first column of each microarray is allocated for gold mirrors, which are visible in Figure 2A–C as bright and whitish squares. In this study, we chose grating order‐coupled nanogaps (GONGs) as the resonator structures for the metasurface because nanogaps and nanorods are one of the most established antenna designs due to their excellent performance improvement in sensing and spectroscopy, as well as for reproducible and scalable fabrication. In particular, we leveraged the linear arrangement of nanorods featuring nanogaps in the x‐direction to achieve high sensitivity in a reliable manner.^[^ ^44^ ^]^ The SEM image of the GONGs design with the parameters of – nanorod length, L = 1500 nm, width, W = 100 nm, y‐periodicity, P_y_ = 3200 nm and the x‐axis gap between the nanorods, G = 80 nm is shown in Figure 2D. These parameters are chosen to have a plasmonic resonance matching with the protein fingerprint regions of 1500–1700 cm^−1^, which is obtained through simulations using a frequency‐domain solver. During this optimization step, for maximizing the sensitivity, we also took into account practical considerations such as target protein accessibility and fabrication reliability (Detailed results and explanations are provided in Section S1 and Figure S1, Supporting Information). The chosen parameters from the simulation procedure are then used in the fabrication step and validated experimentally by spectral characterization of the optical response in aqueous conditions (see Figure S1 C, Supporting Information).The AFM characterization of the nanorods and the SEM characterization of the SU‐8 microwells are presented in Figure S2 (Supporting Information). To efficiently spot the protein solutions onto micrometer‐sized sample wells, we used a semi‐automated low‐volume piezoelectric dispenser. For illustration purposes, Figure 2E shows the optical image of the 96‐microwell chip with colored buffer dispensed and dried, highlighting the ability to hold the samples spatially confined within each microwell of the microarray chip (real video of a fabricated 96‐microwell sensor is provided in supplemental video S2).

The important parameters governing the capability of the proposed microarray are sensitivity, the amount of required samples, measurement speed, and the number of analyzed samples per chip. To evaluate these, we used Bovine Serum Albumin (BSA) as a model protein. We thiolated three differently designed microarray chips (48‐, 96‐, and 384‐microwells) overnight with a mixture of N‐hydroxysuccinimide (NHS)‐activated carboxyl thiols and OH‐spacer thiols in a 1:9 ratio, forming a uniform monolayer of protein‐capturing NHS esters on the gold surface, which is suitable for highly purified samples used in drug screening applications. Next, we washed the chips and spotted selected microwells with a BSA solution of 50 µM stock concentration and used them as sensing units. The remaining unspotted microwells served as reference units. After incubating the BSA solution at room temperature for ≈2 h, the proteins are covalently captured by the NHS ester molecules close to the nanorods' surface in the sensing units for high sensitivity. To perform in situ SEIRA measurements in an aqueous environment, we integrated the microarray chips with 3D printed microfluidic components consisting of a flowcell (white part in Figure 2F) and filled with buffer (PBS 1x) solution. This optofluidic microarray is flipped upside down, and the individual units (sensing, reference, and gold mirrors) are probed from the CaF_2_ side in reflection mode under an FTIR microscope, as shown in Figure 2F. Figure 2G presents the reflectance spectra from reference (dotted line, R_0_) and sensing (solid line, R) units. Both spectra show excitation of the plasmonic resonance around the protein amide range with a peak at ≈1600 cm^−1^. The large dip in the curves indicates the presence of water as it has strong absorption bands peaked around 1650 cm^−1^. The solid line (R) additionally contains small dips in the range 1500–1700 cm^−1,^ indicating the presence of protein. To better visualize the enhanced absorption bands of protein in the Amide I and II bands from the sensing units, we calculated the absorbance (mOD) using the formula of −1000*log_10_ (R/R_0_) and plotted the resulting spectra in Figure 2H. Figure S3 (Supporting Information) and the Experimental Section details the baseline correction method used to obtain the visualized data.

We first quantified the sample volume required per chip for reliable protein detection and adequate binding on the sensing units. Figure 2I is a dot plot denoting the minimum sample volume (just enough to cover the sensing unit) and the maximum sample volume used to cover the entire microwell without overflowing. Intuitively, the highest sample volume is required in the 48‐microwell chip, 200 nL, with a minimum requirement of 89 nL, just enough to cover the plasmonic sensing units. We can sufficiently reduce the sample volume by almost a third in the 96‐microwell chip to 70 nL and even further to only 22 nL for the minimum coverage. The 384‐microwell chip fares quite well in reducing the sample requirement down to a mere 8 and 2 nL for maximum and minimum volume, respectively. In comparison, the traditional multiwell plates need sample volumes orders of magnitude higher (≈50–360 µL/well) for detection. The corresponding absorption profiles of these different sample volumes are presented in Figure 2J. Since our interest lies in the protein structural analysis, from here on, we present only the Amide I band that envelopes the secondary structural band information. We can observe a direct correlation between the protein sample volume and the strength of the absorption signal. The 48‐microwell chip has a maximum absorption strength of ≈40 mOD corresponding to 200 nL, amounting to 665 ng of the protein and ≈32 mOD for 296 ng, followed by absorption retrieved from the 96‐microwell chip from 233 ng (≈24 mOD for 70 nL) and 73 ng (≈17 mOD, 22 nL). We can observe that the signal is slightly lower when only minimum volume is dispensed in all 3 different designs compared to filling the microwells, since a lesser amount of protein is present in the volume. Even then, it is enough to get a considerable absorption strength, as can be seen from the signal strength (≈10 mOD) from only 7 ng of BSA, which can be increased to ≈13 moD with 27 ng of BSA in the maximum capacity of the 384‐microwell sensor chip.

It is worth noting that all these measurements shown above are raw spectra without any smoothing or averaging, taken from a 32‐scan measurement, lasting only ≈17 s, indicating the fast performance for retrieving a good signal. To better quantify this, we measured the signal‐to‐noise ratio (SNR) of our different microarray sensors with the square root (SQRT) method, which is detailed in the Experimental Section. We first calculated the noise level (BG) corresponding to the absorbance signal calculated from a reference unit. To better quantify the signal strength, we define the reliable signal level as at least three times the noise level. We then calculated the SNR for different measurement times (correspondingly, different numbers of scans) as it plays a crucial role in collecting sufficient photons to the detector for a good signal. The results are presented in Figure 2K. The SNR increases with an increase in measurement times in all three chips. The 384‐microwell chip has the lowest, and the 48‐microwell chip has the highest SNR in every scan because the sensing area and, correspondingly, the photon count are increased in the latter case. We observe that even in the lowest of the two scans lasting 1–2 seconds, the signal is still above the corresponding 3 x BG signal (horizontal dotted lines) for all the microarray designs. In the higher scans of 128 and 512, lasting ≈1 and ≈5 min, respectively, the SNR values of the 96‐microwell sensor are on par with those of the 48‐microwell sensor.

Next, we evaluated the signal variations along the replicates of the protein microwells within the same microarray chip design. Figure 2L shows the absorption profiles taken from three different BSA‐coated microwells per chip with the maximum volume. We clearly observe that the absorption strength and peak shape obtained for three replicate microwells in each of the 48‐, 96‐, and 384‐microarray sensor chips are similar, emphasizing the reproducibility of sample measurements within the same chip using our sensor. We also characterized the Limit of Detection (LoD) and concentration response of our sensors. We spotted the 48‐, 96‐, and 384‐microarray sensor chips with BSA at a wide range of concentrations, from 100 pg mL^−1^ to 20 mg mL^−1^, in triplicate for each chip design, and the corresponding obtained absorbances are plotted in Figure 2M. Even at the lowest concentration of 100 pg mL^−1^, the 96‐well microarray sensor demonstrates a strong signal of ≈1 mOD, while the 48‐well microarray sensor achieves an even higher absorbance of around ≈2 mOD, indicating excellent sensitivity. In contrast, the 384‐well microarray chip shows a lower response, with absorbance values well below 0.5 mOD, which is not significantly above three times the reference noise level, indicating a lower sensitivity in this higher‐density format. The LoD of the 384‐microarray chip is around ≈1 ng mL^−1^. All of the microarray sensors exhibit a broad dynamic range spanning over eight orders of magnitude, with signals approaching saturation near 10 mg mL^−1^ and above, demonstrating their capacity to quantify protein concentrations across a wide physiological and experimental range. Finally, we experimentally tested the stability of our microarray sensors upon long‐term storage after surface functionalizing the chips. The results, shown in Section S4 and Figure S4 (Supporting Information), indicate good stability even under non‐ideal storage conditions, including constant contact with moisture, leading to gradual hydrolysis of the thiols, thereby compromising their capability to bind.

### 2.3 Drug Screening Based on Structural Protein Biomarkers for Neurodegenerative Diseases

**Figure 3:**
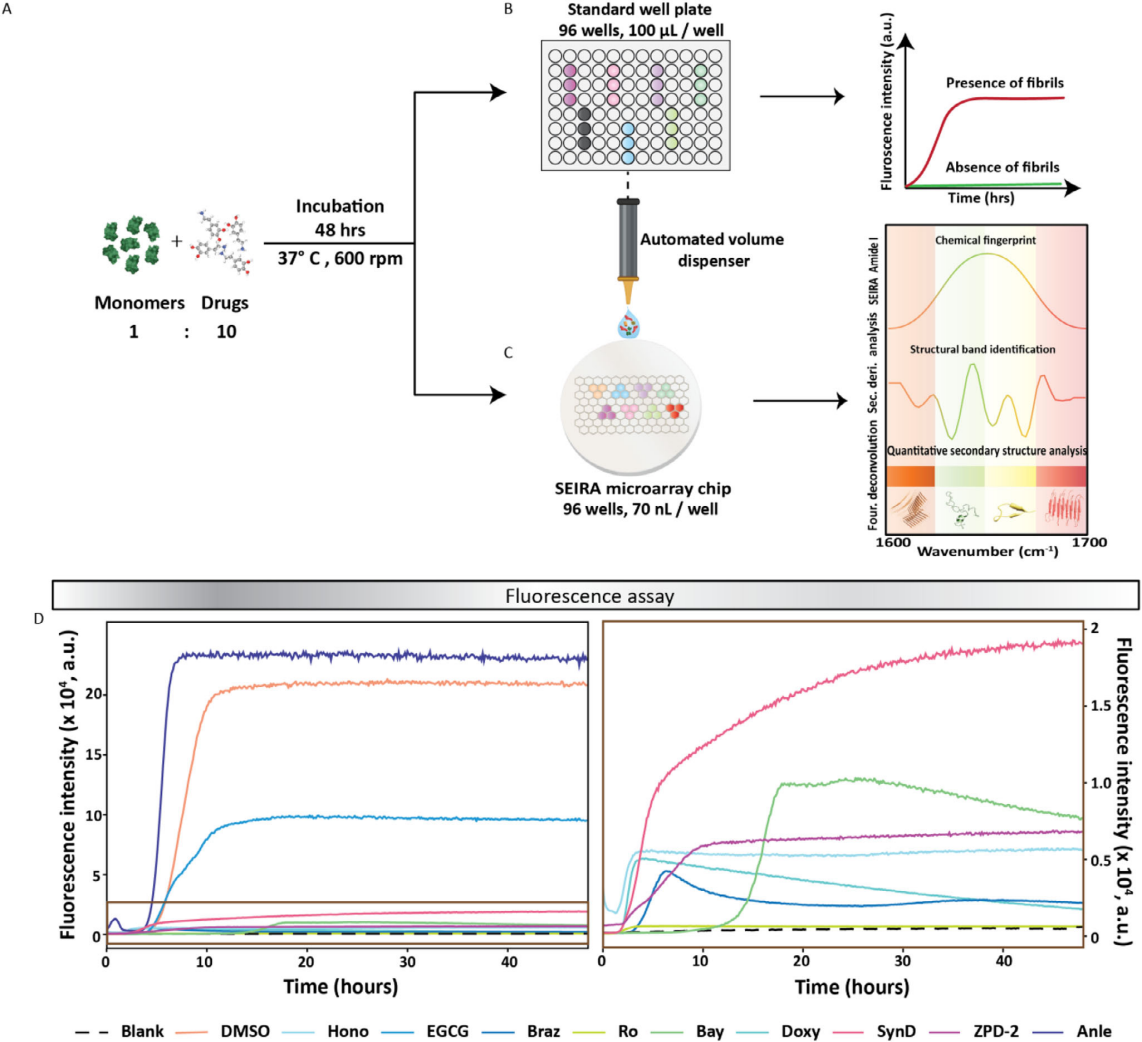
Experimental workflow for the drug screening used in the study. A) Drug incubation assay with conditions. B) ThT‐based fluorescence measurements in the 96‐multiwell plate. C) HT‐SEIRA measurements with the 96‐microwell microarray chip. D) ThT assay results of the drug incubation experiments using nine drug compounds.

After demonstrating the fabrication and performance validation of the microarray sensors, we investigated their suitability for drug screening applications. For this purpose, we chose aSyn as a model protein because it is a well‐characterized protein with highly reproducible preparation and structural analysis, ensuring accuracy in our study. Moreover, aSyn has been recently validated as a definitive structural biomarker for PD, with numerous clinical trials and research efforts actively focusing on targeting aSyn to develop disease‐modifying drugs, further reinforcing its relevance in developing high‐throughput drug screening platforms.^[^ ^53^ ^]^ Consecutively, we selected nine drug compounds listed in **Figure** **3**, which have been shown to modulate the aggregation of aSyn and other amyloid‐forming proteins via distinct mechanisms. Figure S5 (Supporting Information) shows the chemical composition of the chosen drug compounds. We classified these drugs into three categories. The first category is the naturally derived small molecule compounds known for their neuroprotective and anti‐inflammatory properties – Honokiol (herein referred to as Hono),^[^ ^54^ ^]^ Epigallocatechin gallate (EGCG),^[^ ^55^, ^56^, ^57^ ^]^ and Brazilin (herein referred to as Braz).^[^ ^58^, ^59^, ^60^ ^]^ The second category is the class of 3 compounds that are known as Aβ aggregation inhibitors – Ro 90–7501 (herein referred to as Ro),^[^ ^61^, ^62^ ^]^ BAY‐K‐8644 (herein referred to as Bay)^[^ ^61^, ^63^ ^]^ and Doxycycline hyclate (herein referred as Doxy).^[^ ^64^, ^65^, ^66^ ^]^ The final category is the group of compounds extensively studied as aSyn aggregation inhibitors and fibril disintegrators – SynuClean‐D (herein referred to as SynD),^[^ ^67^, ^68^ ^]^ ZPD‐2,^[^ ^69^ ^]^ and Anle‐138b (herein referred to as Anle).^[^ ^70^, ^71^, ^72^ ^]^ We chose these small molecules because they are among the leading drug candidates, with EGCG, SynD, ZPD‐2, and Anle being the most extensively studied compounds for their misfolding inhibiting effects on aSyn with significant clinical relevance; for example, Anle is currently in a Phase 1a trial.

Figure 3A–C shows the experimental workflow employed in this study. We focused on understanding whether the chosen drugs can inhibit the aggregation of native monomeric aSyn. We incubated the aSyn monomers and different drugs in the ratio 1:10 in a conventional 96‐multiwell plate setup for 48 h at 37 °C, continuously shaking at 600 rpm (Figure 3A), together with the ThT dye for doing correlation with the fluorescent assay. The ThT assay gives information on the presence of fibrils with a corresponding increase in fluorescence intensity; otherwise, the fluorescence signal remains at a background level (Figure 3B). We opted for this method as it is the most common assay for studying aggregation kinetics, and we employed it as a reference for our SEIRA measurements. We chose a 96‐multiwell plate because ThT aggregation and seeding assays are optimized for 96‐multiwell plates and are widely used in drug screening due to their standardized protocols, ease of handling, and compatibility with common laboratory equipment. Each sample had three replicates with 100 µL/well, the minimum amount of volume required for acquiring sufficient ThT fluorescence signal. After the drug incubation for two days, each sample (after combining the replicates) is spatially bioprinted onto individual microwells in our thiolated microarray sensor chip using a piezoelectric dispenser. After incubating our chip with these protein mixtures at room temperature for ≈2 h, we integrated the chip with the microfluidics for the in situ HT‐SEIRA measurements (Figure 3C). Here, we used the 96‐microwell sensor chip as it can provide a direct comparison with the 96‐multiwell plate used in fluorescence measurements under identical throughput and assay conditions. While our platform supports 48‐, 96‐, and 384‐microwell designs without requiring changes to instrumentation or protocols, the 96‐microwell format provided the optimal balance of sensing performance, throughput, and relevance to widely adopted drug screening workflows.

The SEIRA measurement results are analyzed in three levels (Figure 3C right panel). The first level retrieves the Amide I absorption band to get an insight into the chemical fingerprint of each sample. The second level involves postprocessing the Amide I band to reveal the secondary structural information by performing a mathematical second derivative analysis. This gives us an unbiased overview of the structural motifs present in the sample based on the peak of the negative bands, providing a semi‐quantitative correlation to the value of the negative peak. Based on those peak values, as a third level, we perform the Fourier self‐deconvolution (FSD) method, which is the standard and well‐established method to calculate the quantitative contribution of different secondary structural motifs present in the protein mixture by deconvoluting their overlapping absorption bands through curve‐fitting.^[^ ^73^ ^]^ This way, we can perform a comprehensive structural profiling of all the samples using only the Amide I band. The detailed methodology and the procedure employed to perform the absorbance retrieval, second derivative analysis, and FSD for quantitative secondary structure determination are explained in the Experimental Section under FTIR data analysis and absorbance calculation, and Secondary structure analysis.

Figure 3D (left) shows the ThT assay results obtained for the aSyn monomers sample treated with the 9 drugs. We used the mixture of aSyn and DMSO (solvent used for drug elution) as the negative control, and we observed that the ThT signal increased over time as the fibrillization progressed. The mixture samples with EGCG and Anle compounds also showed increased signal intensities. The remaining samples showed decreased values, implying the inhibition of fibrillization/aggregation. Figure 3D (right) shows the zoomed‐in plots of those samples. Some have reduced signal intensities but produce similar aggregation curves, even if it is 10–20 times lower in intensity. The sample with Ro shows a similar response to that of the blank signal containing only ThT and buffer, indicating either effective inhibition of aSyn monomer aggregation or the presence of a mixture of conformational or aggregation states with reduced fibril presence.

Next, we proceeded with HT‐ SEIRA measurements following the same procedure as in Figure 2 (detailed in the Experimental Section). The samples are spotted onto the 96‐microarray sensor chip with replicates across three microwells. In addition to the ten samples (one negative control and nine drugs) from the ThT fluorescence assay, we spotted pure aSyn monomers and pure aSyn fibrils for a comprehensive reference and comparison of secondary structure content. We then performed the SEIRA measurements of the samples using an FTIR microscope and measured the reference and sample microwells with 128 scans, lasting a minute per measurement. We retrieved the chemical fingerprint absorbance in the Amide range (1500‐1700 cm^−1^) from each sensing unit (Detailed procedure in the Experimental Section under FTIR data analysis and absorbance calculation). For better clarity, we only show the Amide I band (1600‐1700 cm^−1^) in the following figures, as it is the relevant amide band for structural analysis.

**Figure 4:**
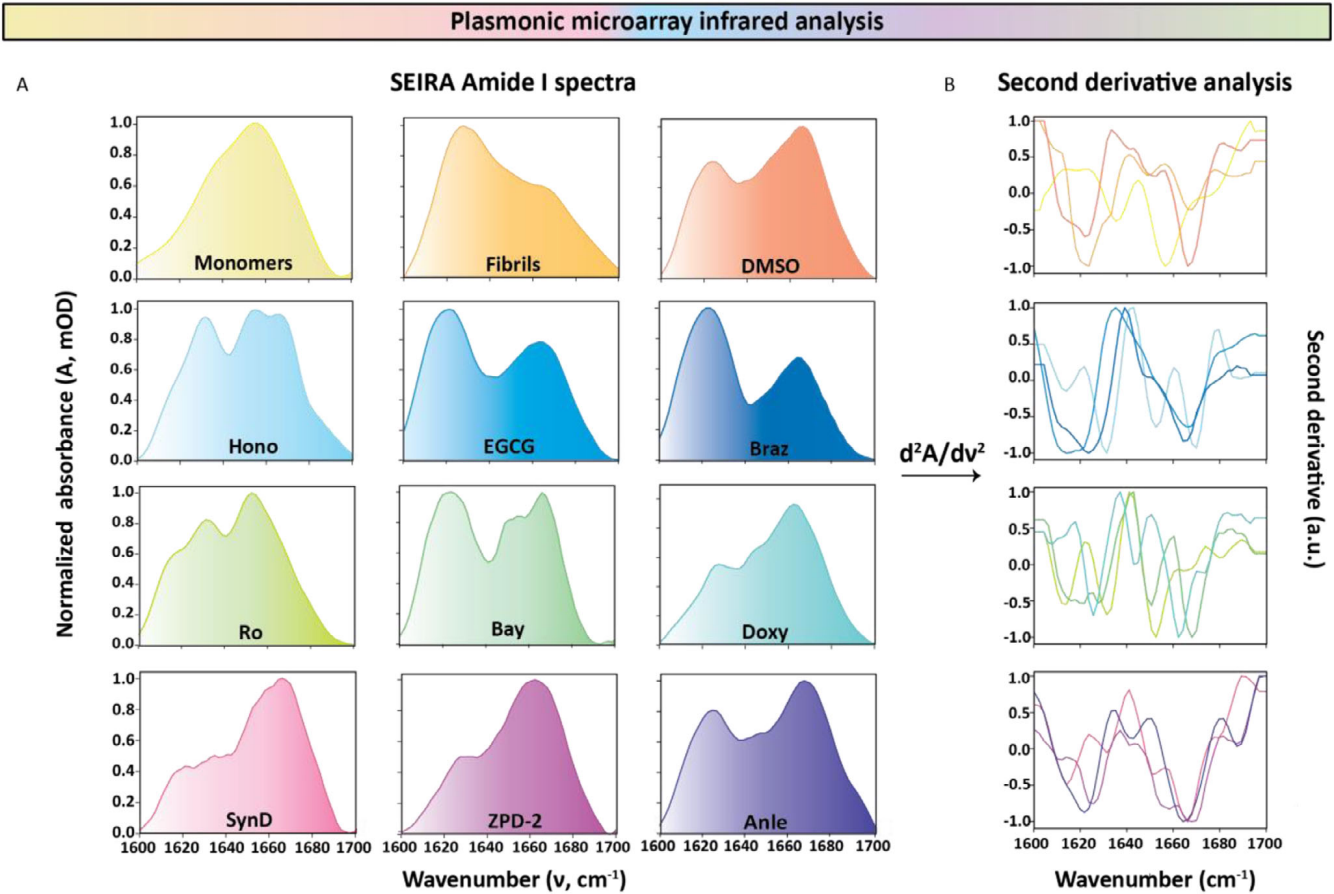
Plasmonic infrared microarray analysis of the drug screening experiments showing A) the Amide I spectra retrieved from each protein‐drug mixture and B) their corresponding second derivative analysis.

**Figure** **4**A shows the averaged Amide I spectra retrieved from three replicates of each sample as the first level of the IR analysis. The first row shows the results for controls of SEIRA measurements, which are pure monomers, pure fibrils, and the negative control (aSyn incubated with DMSO in ThT assay). We can already observe apparent differences in their characteristic peaks. The monomer sample has a peak around 1650 cm^−1^, indicating the abundant disordered structure. In comparison, the fibril sample has a prominent peak around 1625 cm^−1^, showing the dominance of β‐sheets presence, with an off‐peak shoulder at 1670 cm^−1^, showing the presence of β‐turns. The negative control sample clearly shows an off‐shoulder peak at 1625 cm^−1^, indicating the presence of parallel β‐sheets and the formation of fibrils in the absence of drugs. This correlates with the ThT assay showing a high fluorescence signal for the DMSO‐treated sample. We can infer from these results that all of the studied drug mixtures show clear differences in their Amide I spectra compared to pure monomers, which is the starting point in the drug incubation assay. At the same time, they do not reproduce the exact spectra of pure fibrils, indicating the formation of different structural forms due to drug interaction and the possibility of different modes of drug action.

Even within the same classes of drugs, we observe differences in the measured spectra regarding the band shape and nature of peaks. In contrast, in the ThT assay, differences in fluorescence signal intensities are observed. For example, the samples treated with EGCG and Braz show a predominant peak in the parallel β‐sheets range; however, in the ThT assay, they produce contrasting results, with the latter showing very weak signals. The Hono‐treated sample has nearly equally contributive peaks in the lower and higher wavenumber ranges of the Amide I band. The Bay‐treated sample gave a profile similar to the Hono. The Ro‐treated sample shows a clear peak around 1625 cm^−1^, indicating the presence of β‐sheets structures, contradictory to the ThT assay result, with the signal value close to the blank. The Anle‐treated sample, which had a very high ThT value, produced the Amide I band with peak features similar to that of the DMSO‐treated sample. Whereas the samples treated with the rest of the drugs (SynD, Doxy, and ZPD‐2) show minimal off‐shoulder peaks in the parallel β‐sheets range compared to the higher wavenumber ranges at around 1625 cm^−1^. These results demonstrate the superiority of the HT‐ SEIRA as it is able to detect differences that are masked by the interference/quenching of drugs with the ThT fluorescence (Figure S6, Supporting Information) and provides more mechanistic insight into the mode of action of the drugs.

The distinctive response from different drugs necessitates a quantitative secondary structure analysis to get better insights. For this purpose, we performed the second derivative analysis of the obtained Amide I spectra, as shown in Figure 4B (Detailed procedure in the Experimental Section under Secondary structure analysis). This second‐level analysis gives a qualitative overview of different structural forms present in the samples. Correlating with the Amide I band, the second derivative analysis produces distinct curves for each sample. The second derivative plots revealed the distinct bands present in each mixture with better clarity. Every sample except pure monomers reveals a peak in the parallel β‐sheets range in different intensities. The DMSO and Anle‐treated samples have a significant negative peak at ≈1625 cm^−1^, showing a correlation with the ThT assay, indicating the presence of fibrils. Even though the Ro sample had a weak signal in the ThT assay, it shows two prominent peaks in the parallel β‐sheets range of 1605–1640 cm^−1^, necessitating further validation steps. EGCG‐ and Braz‐treated samples have the maximum negative peak in the parallel β‐sheets region, suggesting the maximum presence of the aggregates in the mixture. However, the corresponding ThT assay signal of the latter contradicts this finding. SynD‐treated sample, on the other hand, has the lowest negative peak value at ≈1625 cm^−1^, and samples treated with Doxy, Hono, and Bay have high parallel β‐sheets peaks. However, the fluorescence signal of the former was much higher than the rest of the samples.

**Figure 5:**
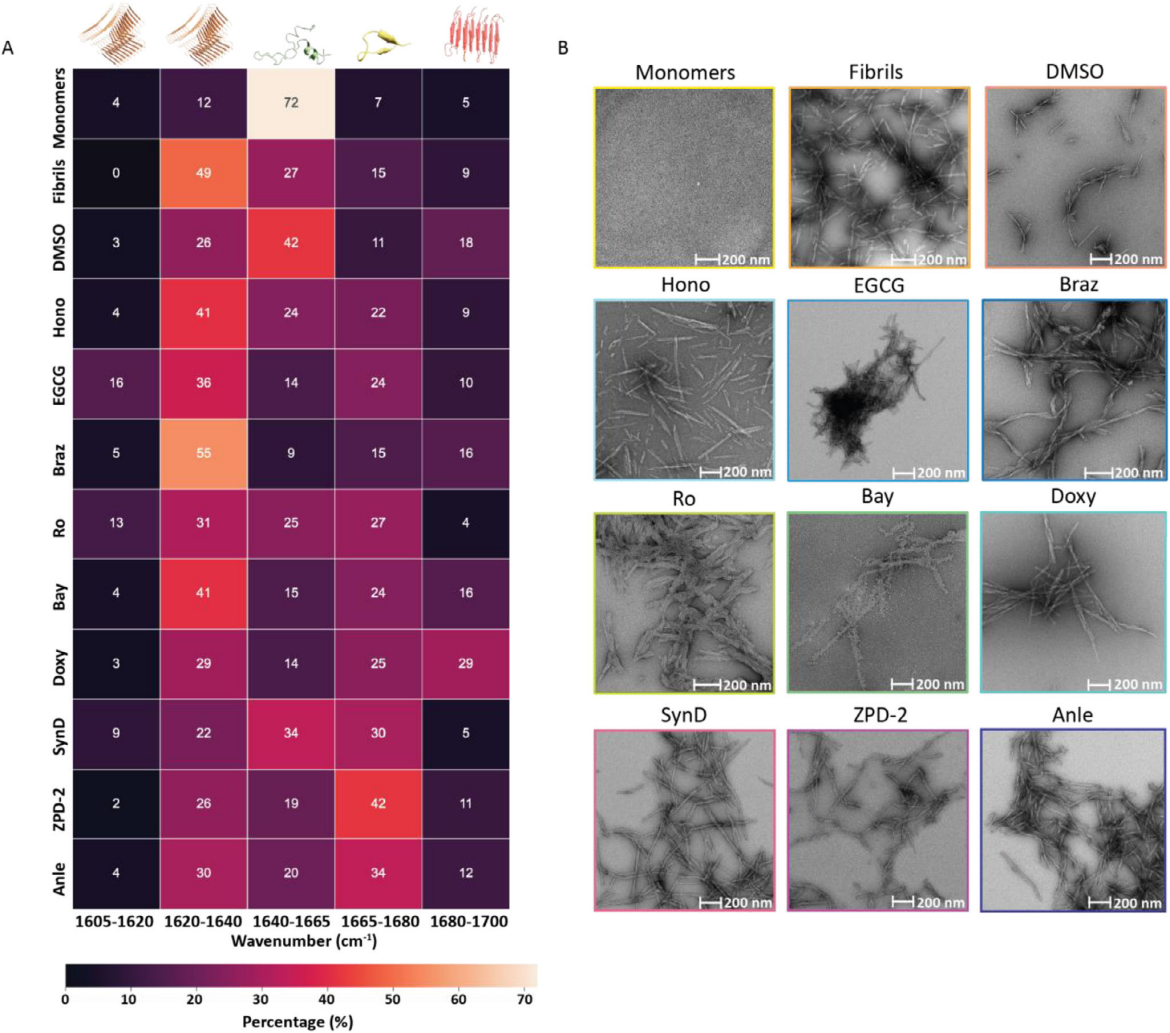
Quantitative structural analysis of drug incubated samples A) Heatmap providing percentages of distinct secondary structural motifs in the protein‐drug mixture. B) TEM images of the protein‐drug mixtures for correlation.

With such contrasting results observed between the fluorescence assay with the Amide I band spectra and the second derivative analysis, a comprehensive quantitative analysis is essential to reveal the accurate secondary structure forms in the protein‐drug mixture to correlate the ThT assay with the infrared measurements. The quantitative data of different secondary structural motifs in each sample are extracted by performing FSD based on the peak position information obtained from the second derivative analysis (Detailed procedure in the Experimental Section under Secondary structure analysis). The results from this third‐level analysis are displayed in **Figure** **5**A (also Figure S7, Supporting Information). The heatmap shows the percentage of different secondary structure motifs – parallel β‐sheets, disordered/α‐helix, β‐turns, and antiparallel β‐sheets, present in the samples. The map reveals a distinct structural map for each sample. To correlate our findings, we performed Transmission Electron Microscope (TEM) imaging on the samples at the same endpoint used for the SEIRA analysis (Figure 5B).

From the SEIRA measurements, we calculated that the pure monomer sample has the highest percentage of disordered structure (72%) and the lowest total β‐sheets content (21%). Since aSyn monomers are small‐sized proteins lacking ordered structure, they cannot be imaged in TEM. All the other samples show the presence of fibrils in the TEM images. The pure fibril sample shows more than half of its structural presence in β‐sheets (58%), with the disordered motif presence reduced to 27%. The corresponding TEM image showed a uniform population of twisted double‐stranded fibrils. The protein sample treated with Braz revealed the presence of fibrillar aggregates with the highest contribution of β‐sheets (76%). The corresponding TEM image shows long, intertwined fibrillar species, explaining the abundance of β‐sheets. Hono‐ and Bay‐treated samples have a similar parallel β‐sheets presence with slight differences in their disordered and antiparallel β‐sheets strength. Even with these slight changes, we can observe some morphology differences in their corresponding TEM images. The Doxy‐treated sample has the highest antiparallel β‐sheets content of 29% with a similar contribution from parallel β‐sheets. The sample with Ro shows a distinct morphology of fibrils present in TEM, with the infrared structural analysis showing an overall β‐sheets presence of 48%, with the rest shared between disordered and β‐turns. ZPD‐2‐ treated sample has the most abundant presence of β‐turns (42%) with 39% of β‐sheets, and its TEM images show scattered, few populations of small fibril species. EGCG also has a dominant β‐sheets presence (36%), with the TEM image showing the morphology of clustered small fibril species. SynD‐ and Anle‐treated samples also have a similar parallel β‐sheets presence, with Anle having a slightly increased presence of β‐turns and antiparallel β‐sheets, each showcasing similar fibrillar morphology in their corresponding TEM images.

With the exceptional ability of our SEIRA microarray sensor, we were able to thus characterize each sample and quantitatively retrieve their distinct structural map, which was then validated by TEM. Based on these results, we observe that none of the drugs are effective in inhibiting the aggregation of aSyn monomers, as evident by the presence of fibrils in all analyzed samples. Also, the TEM images correlate better with the structural analysis obtained from the SEIRA measurements than with the fluorescence assay results. Strikingly, fluorescence assay gave false negative results in the case of Ro‐, ZPD‐2‐, Braz‐, Doxy‐, and Hono‐treated samples, where there was apparent fibril formation as validated by TEM and SEIRA analysis. This discrepancy may be primarily due to the quenching effects arising from either the sheer presence of drugs in the mixture or the binding of the drug molecules to the pockets of the protein structure, where the ThT molecules need to bind to produce increased fluorescence emission, which is blocked in this case.^[^ ^74^, ^75^ ^]^ At the same time, our negative control experiments indicate that the drug compounds induce no interfering absorbance in the wavenumber range of the Amide I and II bands, indicating no potential drug‐plasmonic substrate interaction that could hamper the protein structural assessment using the SEIRA technique (Section S8 and Figure S8, Supporting Information). We also performed control experiments to evaluate the structural integrity of aSyn protein once bound to our gold surface to observe any potential interference from the localized heating emerging due to the resonance effect. The results presented in Figure S9 (Supporting Information) indicate that the protein structure is unaffected by the long‐term presence of aSyn protein on our sensor surface and by the localized heating experienced by the proteins for a very short time during the microwell measurements.

In addition to structural sensitivity and high‐throughput capability, reproducibility is a critical requirement for any microarray sensor intended for practical applications. We performed a reproducibility study involving multiple independent experimental runs, using different microarray sensor chips and independently prepared sample sets. The results, presented in Section S10 and Figure S10 (Supporting Information), highlight the strengths of our plasmonic SEIRA microarray sensor in terms of its ability to handle numerous replicates, retrieve structural resolution with accuracy, and provide cost‐effective and scalable analysis across multiple experimental runs. We also performed a dose‐dependent study (Section S11 and Figure S11, Supporting Information), providing valuable insights into the structural impact of varying drug concentrations on aSyn aggregation. The results further underline the value of SEIRA‐based secondary structure analysis in pharmacodynamic evaluations and illustrate the utility of our plasmonic microarray sensor for high‐throughput, concentration‐dependent drug screening in NDDs research.

### 2.4 Characterization of Different Oligomeric forms in the Aggregation Pathways using Plasmonic SEIRA Microarray Sensor

Apart from exogenous drug compounds, endogenous biochemical factors play a crucial role in modulating the aggregation behaviour of aSyn and influencing the progression of PD and other NDDs. In particular, Dopamine (DA), a key neurotransmitter, and 4‐hydroxy‐2‐nonenal (HNE), a by‐product of oxidative stress and lipid peroxidation, have been shown to significantly alter the aggregation pathway of aSyn.^[^ ^76^, ^77^, ^78^ ^]^ Unlike the small molecule drugs tested earlier, these molecules induce the formation of off‐pathway, soluble oligomeric species, which are structurally distinct from the β‐sheets‐rich‐fibrils typically detected in conventional assays. As a result, the ThT fluorescence assay, which is optimized for detecting fibrillar aggregates, fails to capture the presence and structural characteristics of these toxic oligomers.

**Figure 6:**
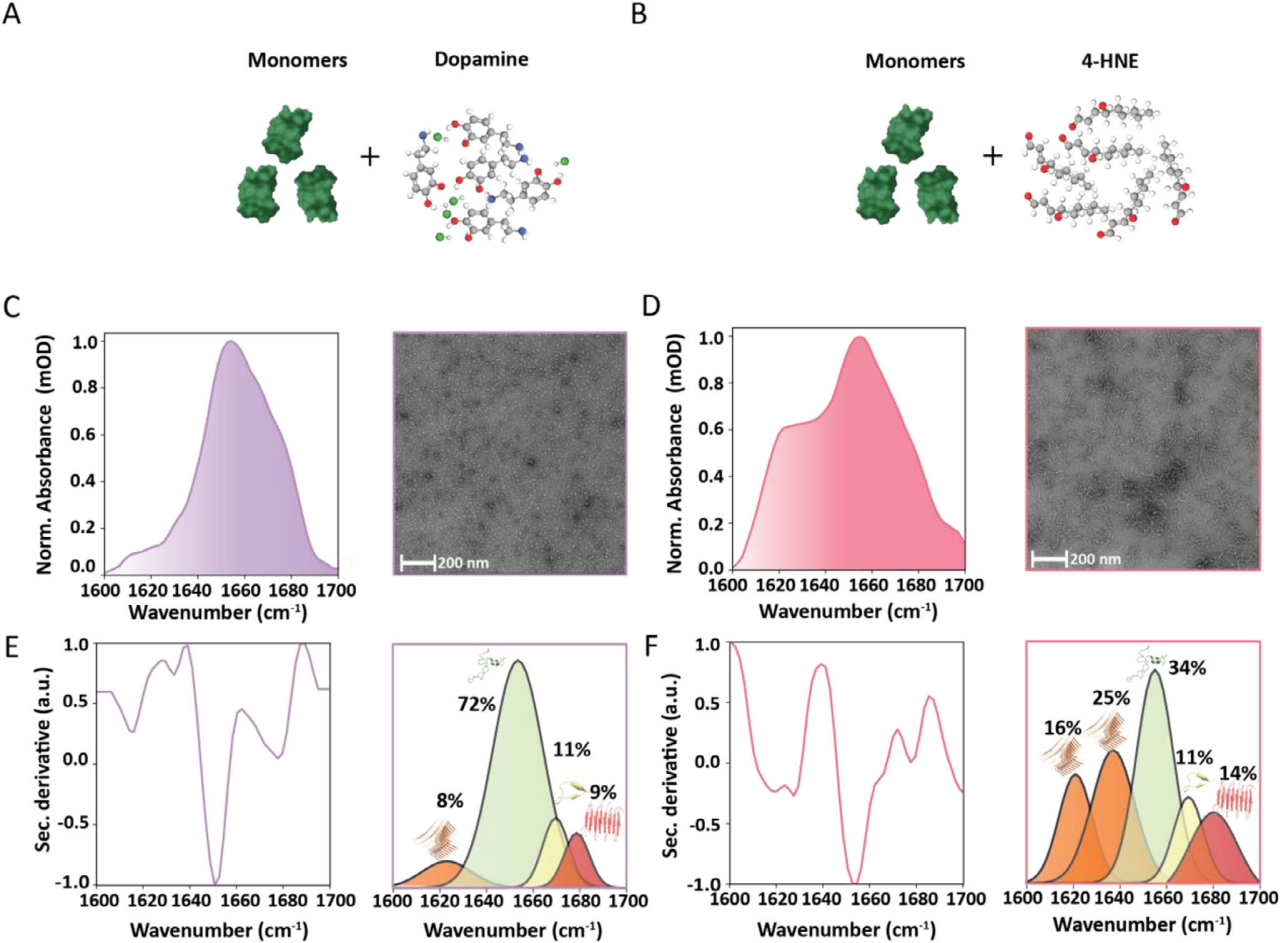
Characterization of different oligomeric forms in the drug‐treated samples. Incubation of aSyn monomers with A) Dopamine (DA) and B) HNE. C) Amide I spectra and TEM image of DA‐oligomers. D) Amide I spectra and TEM image of HNE‐oligomers. Second derivative and quantitative secondary structure analysis of E) DA‐oligomers and F) HNE‐oligomers.

To evaluate the capability of our microarray sensor to capture the diverse conformational characteristics of such off‐pathway structural species, we incubated DA and HNE with aSyn monomers separately (detailed procedure in the Experimental Section), as shown in **Figure** **6**A,B. We performed SEIRA measurements and TEM imaging of their end products, and their results are shown in Figures 6C,D. The TEM images show the oligomeric formation of aSyn with very distinct morphologies for the final aggregation product when interacting with DA and HNE. The DA‐induced oligomers have heterogeneous but small spherical metrology (Figure 6C right). Meanwhile, the HNE‐induced oligomers show a distinct and uniform curvilinear morphology (Figure 6D, right), consistent with previous reports.^[^ ^79^ ^]^ The structural state of these oligomeric forms is investigated using SEIRA measurements by retrieving their Amide I band (detailed procedure in the Experimental Section under FTIR data analysis and absorbance calculation). Interestingly, the Amide I band of DA‐oligomers has a predominant peak at ≈1658 cm^−1^ (Figure 6C left). In contrast, the HNE‐oligomers produce a maximum peak at 1660 cm^−1^ with an off‐shoulder peak around ≈1620 cm^−1^ (Figure 6D left), indicating the presence of parallel β‐sheets. Through further structural evaluation using second derivative analysis and FSD (detailed procedure in the Experimental Section under Secondary structure analysis), we estimated that the DA‐oligomers have highly dominant disordered structures with 72% absorption in the wavenumber range of 1640–1665 cm^−1^ with a β‐sheets presence of merely 17% (8% parallel and 9% antiparallel). At the same time, the HNE‐oligomers have more than half of their structure in β‐sheets form (55% – 44% parallel and 14% antiparallel), with the disordered motif reduced to 34%. In both cases, the fluorescence measurements have failed to give any detectable signal (Figure S12, Supporting Information). In contrast, our results are aligned with the previous literature, and we further validated our results using CD spectroscopy, as presented in Figure S13 (Supporting Information).^[^ ^79^ ^]^ This demonstrates the unique strength and reliability of our HT‐SEIRA microarray sensor in handling complex drug screening assays across various conditions in a high‐throughput manner, highlighting the critical importance of integrating our proposed method into the drug screening workflow.

## 3 Conclusion

In conclusion, we present a powerful nanoplasmonic infrared microarray sensor for secondary structural protein biomarker‐based drug screening in NDDs in a high‐throughput manner. The plasmonic sensing units employ the SEIRA principle to achieve highly sensitive in situ infrared fingerprint measurements by tailoring the nanoantenna design to resonantly match the protein absorption bands. The plasmonically enhanced Amide I band facilitated quantitative structural analysis of proteins with enhanced structural sensitivity, providing comprehensive information on the presence of different secondary structural forms present in the protein‐drug mixture. To add the high‐throughput detection feature, we leveraged the 2D pattern of sensing units by the precise micropatterning of polymeric microwell arrays compartmentalizing the individual nanoplasmonic sensing units, allowing simultaneous measurement and detection of numerous samples on a single chip. We demonstrated the versatility and adaptability of our technique by fabricating different microarray designs with 48, 96, and 384 sample microwells. These microarray designs are miniaturized onto a compact 3 cm‐sized chip, offering significant advancements over the currently used large‐format multiwell plates. We showed the superior protein detection sensitivity of our microarray sensor from sample volumes as low as 2 nL at a high SNR and with a LoD of down to 100 pg mL^−1^, which is orders of magnitude superior to the traditional optical HTS and IR techniques with a sensitivity of a few mg/ml. We demonstrated the application of our microarray sensor chip for drug screening in NDDs by assessing nine different drug compounds for their inhibiting effect on the aggregation of aSyn protein from healthy monomers to pathological fibrils. The combination of nanoplasmonic SEIRA detection and microwells enabled highly sensitive structural detection of proteins in a label‐free and high‐throughput manner using low sample volumes, making it suitable for integration into a drug screening workflow. We retrieved the structural map of each drug‐treated sample and quantitatively identified different secondary structural forms from plasmonically enhanced infrared absorption spectra with excellent reproducibility. Compared to the gold‐standard ThT fluorescence assay, which merely gives a semi‐qualitative output about the presence of fibril formation, our method provides comprehensive structural information extracted from a single protein Amide I band. Furthermore, our microarray sensor reliably extracted the structural fingerprints when the protein‐compound (DA and HNE) interaction resulted in the formation of oligomeric aggregates with distinct morphologies, whereas the ThT assay failed to detect them. We were able to provide a fully quantitative composition of distinct structural motifs present in the DA‐ and HNE‐oligomers, which has been missing from previous studies. These results demonstrate the advantages of our microarray sensor in relevant performance metrics in comparison to the existing technologies used for secondary structure analysis (see Section 14 and Table S1, Supporting Information for comparison).

Overall, thanks to the precise on‐chip integration of sensitive nanoplasmonic IR sensors with small‐volume fluidic microwells, we were able to provide a first‐of‐its‐kind successful demonstration of an ultra‐compact SEIRA microarray sensor for high‐throughput drug screening applications. We exhibited its ability to monitor clinically relevant drug effects on protein aggregation pathways – capabilities beyond the reach of standard assays like ThT. Concurrently, our findings highlight the power of using structure‐based screening platforms to identify drugs that modulate the aggregation of aSyn and other NDDs‐proteins while at the same time providing initial insight into their mode of action. The ability to conduct this assay in a high‐throughput format with excellent repeatability using a minimal amount of samples addresses limitations that have precluded the use of other commonly used assays, including the limited amount of proteins available to conduct the assay and screening of minute amounts of protein aggregates isolated from cells or human brain.

As an outlook, our introduced device can be straightforwardly adapted to immobilize and study any other NDDs‐related structural biomarker proteins, such as tau, Aβ, and TDP‐43, using the generalized surface chemistry employed in this study, without requiring any sensor reconfiguration. Our microarray sensor simultaneously offers direct compatibility for multiplexed in vitro screening of different NDDs protein biomarkers and broader NDDs research, thanks to the high‐throughput capabilities realized using our microarray design. We envision that it could be integrated into the drug discovery pipeline in the lead optimization (HT‐ in vitro screening) stages for NDDs. Given the availability of infrared micro‐spectroscopy systems in pharmaceutical research, the cost and logistics of such an integration are feasible.^[^ ^80^ ^]^ In terms of future technology improvements, recent developments of compact and highly sensitive QCL‐based IR imaging systems offer simpler and user‐friendly interfaces, making infrared measurements adaptable even for non‐technical specialists. In combination with fully automated, low‐volume sample dispensers, such IR systems can accelerate the screening process. For scalability, a key consideration is the cost‐effective fabrication of our nanoplasmonic SEIRA microarray sensors on a wafer scale. Even though, at the current stage, we employed a research‐grade, low‐throughput E‐beam lithography process at the chip scale with reasonably low costs, our sensor design is translatable to the wafer scale production using high‐throughput E‐beam, deep ultraviolet lithography, or nanoimprint lithography techniques available in industrial foundries.^[^ ^81^ ^]^ This transition to wafer‐scale production would significantly enhance manufacturing efficiency while further reducing costs, bringing our proposed technology closer toward large‐scale and practical applications in biosensing. This could also be complemented by the adaptation of CMOS‐compatible plasmonic material, such as Aluminum (Al), for nanoantennas.^[^ ^82^, ^83^ ^]^ To further increase the spectral information content, new metasurfaces can be designed to simultaneously access Amide I and III protein bands for better structural resolution or cover also the absorption bands of the compounds to get chemical data of the used drugs. In terms of spectral data analysis, the current device scheme can be combined with our previous work on real‐time ImmunoSEIRA sensor and AI algorithm to extract time‐resolved data on aggregation progression and provide the capability of quantitative discrimination of different structural forms present in the aggregation mixture.^[^ ^84^ ^]^ This can help us to gain new insights into different molecular pathways of aggregation and the mode of action of drugs.

## 4 Experimental Section

### Fabrication of the Plasmonic Metasurfaces

CaF_2_ chips measuring 3 cm in diameter and 500 µm thick (Crystran, UK) were used as substrates. The chips were cleaned using the RCA1 protocol (NH4OH: H2O2: H2O = 1:1:5), followed by rinsing with acetone and isopropanol (IPA). Subsequently, the chip was spin‐coated with a double layer of poly(methyl methacrylate) (PMMA). Before E‐beam lithography, a 10 nm gold layer was deposited as a conductive layer on the chip with DC Magnetron sputtering. Nanorod arrays with dimensions of 1500 nm in length, 100 nm in width and height, with a y‐periodicity of 3200 nm, and an 80 nm gap between the nanorods in the x‐direction are patterned using E‐beam lithography (Raith EBPG5000+) with a 5 nm resolution, and gold mirrors are created with a 100 nm resolution using a 100 keV electron beam. 48‐microwell, 96‐microwell, and 384‐microwell sensors were patterned as 6 × 8 (rows x columns) with individual array sizes of 750 × 750 µm^2^, in 8 × 16 with array size of 500 × 500 µm^2^, and 16 × 24 with array sizes of 250 × 250 µm^2^, respectively. The microarray design also contained four alignment markers written with 5 nm resolution for the microwell patterning later. The gold layer was removed using a wet etching process with a KI + I2 solution, followed by development with MiBK: IPA = 1:3 solution. 5 nm of Chromium and a 100 nm layer of Au were deposited by electron beam evaporation, followed by a lift‐off process with acetone to realize the nanorod structures. SEM characterization was done to confirm the successful fabrication of the structures.

### Fabrication of Microwells

Microwell fabrication was done as a second layer of lithography on the chips with the plasmonic metasurface design. The chips are treated with Oxygen plasma for surface dehydration, followed by spin coating of SU‐8 negative epoxy photoresist with a thickness of ≈70 µm (SU8 3050, Kayaku Advanced Materials, Inc.). The resist‐coated chip was soft‐baked before the exposure. To precisely align the honeycomb patterns to enclose the plasmonic units, an aligned exposure step was used. For this, a photomask with the desired honeycomb‐shaped microwell pattern with alignment markers matching the ones on the chip was fabricated using a direct laser writer (VPG200, Heidelberg instruments). The chip was then exposed using the photomask on the mask‐aligner exposure tool (MA6Gen3, i‐line, Süss MicroTec). After post‐exposure bake, the chip was developed with PGMEA solution to reveal the honeycomb‐shaped microwells.

### Drug Incubation and ThT Fluorescence Assay

All the drug compounds used in this study were purchased from Cayman Chemicals and TargetMol (ZPD‐2). The stock solutions were made by dissolving the compounds in DMSO and stored at −80 °C until use. The drug incubation protocol involved making a master mix solution of 400 µl for each drug compound containing 50 µM of aSyn monomers, 500 µM of drug molecules and 50 µM of ThT dye in PBS 1x buffer. Following this, 100 µl of replicates from each sample master mix solution was added to the three individual wells in a black 96‐multiwell optimal bottom plate (Costar), with each well pre‐added with six SiLibeads ceramic beads (Sigmund Lindner) with a diameter of 1.0 to 1.2 mm. The plates following the preparation were sealed with Corning microplate tape and transferred to a FLUOstar OPTIMA plate reader (BMG Labtech). The parameters used were continuous orbital shaking at 600 rpm at 37 °C; ThT fluorescence was monitored every 300 s using excitation at 450 ± 10 nm and emission at 480 ± 10 nm.

### Surface Functionalization Protocol

Before surface functionalization, the microarray sensor chips were washed with ethanol, IPA, and water, followed by Oxygen plasma to make the surface hydrophilic. Afterward, the chips were immersed in a 2 mM thiol mixture containing activated ester (HSC11EG4OCH2COONHS, ProChimia Surfaces) and spacer thiols (HS‐C6‐EG3OH, ProChimia Surfaces) in a 1:9 ratio overnight. The activated ester molecules consist of a thiol group (SH) for covalent immobilization onto gold surfaces (using the strong bonding between gold and sulfur) and a terminal NHS‐ester group that reacts with primary amine groups on proteins, enabling uniform and stable protein immobilization. The molar concentration ratio between activated ester thiols and OH‐spacer thiols was chosen to be 1:9 to minimize steric hindrance and to achieve better binding performance. The microarray sensor chips were finally washed with ethanol for 1 min and dried before introducing the protein mixture.

### Bioprinting of Protein Drug Mixtures

The thiol‐functionalized microarray chip was placed in a petri dish for protein bioprinting using the tool sciFLEXARRAYER S3 from Scienion (piezoelectric noncontact ultralow‐volume dispensing system). The dispensing system is set to have a relative humidity of 66% and an internal temperature of 16 °C to avoid the drying of spotted sample drops. Each protein‐drug mixture after the drug incubation assay is transferred to a 384‐multiwell plate with a volume of 70 µl, forming the probe plate for the spotter tool. Each protein mixture is then dispensed into three microwells as replicates with a drop volume of 450 pL within the corresponding microarray chip. The volumes differed for different microwells, as described in the main text, to fill the microwells. Afterward, the microarray chip was sealed and kept inside the tool with ambient humidity for almost 2 h. After the incubation, the microarray sensor chips are washed with PBS 1x for 1 min and dried to be used for further experiments.

### In Situ HT‐SEIRA Measurements

For the infrared measurements, the sensor chips were placed on a microfluidic component to hold the chip with the design part exposed for PIR measurements. This holder was flipped and combined with another microfluidic flowcell with a reservoir filled with PBS 1 x buffer so that the plasmonic sensing units come in contact with the buffer and measurements could be done from the CaF_2_ side. The in situ HT‐SEIRA measurements were carried out using a Fourier transform infrared (FTIR) spectrometer (Bruker Vertex 70v) coupled to a microscope (Hyperion 2000 IR microscope) with a reflective Cassegrain objective (15x, NA = 0.4), with High power Globar as the light source and liquid nitrogen cooled Mercury Cadmium Telluride (MCT) as the detector. An external polarizer was used to apply incident light polarization parallel to the long axis of the nanorods. A knife‐edge aperture limits the light collection to slightly less than the respective array sizes in different designs. The measurements were done in reflection mode, illuminating the microarray sensor chip from the backside of the CaF_2_ substrate to avoid light propagation through buffer/water and in a purged dry air environment. For collecting data, mirrors were used for normalizing every reflectance spectra measured from the units. All the reflectance spectra of the mirrors and the sensing units were done with 32 scans for the results in Figure 2 unless otherwise mentioned and 128 scans (≈1 min) for the drug screening experiments and secondary structural analysis with one measurement per microwell.

### FTIR Data Analysis and Absorbance Calculation

The protein absorbance in the range 1500–1700 cm⁻¹ was calculated by dividing the reflectance spectra after protein binding (R) against the reference microwell spectra without any protein (*R*_0_). The differential absorbance spectrum was then retrieved using the formula ‐1000*log10(R/R_0_). A baseline correction was performed using the Asymmetric Least Squares (ALS) Smoothing algorithm described by Eliers et al.^[^ ^85^ ^]^ using the smoothness, asymmetry, and number of iterations parameters as 10^4^, 0.001, and 10, respectively. All the data presented in the main figures were the baseline corrected spectra for better visualization and analysis. The SNR shown in Figure 2 was calculated using the SQRT method using the formula SNR = (Abs_1650_ – Abs_1950_)/sqrt(Abs_1950_).

### Secondary Structure Analysis

Secondary structure analysis follows the protocol from Yang et al.^[^ ^73^ ^]^ The reflectance spectra were collected with 128 scans per measurement and 4 cm^−1^ spectral resolution. After baseline correction, the retrieved absorbance spectra from the individual protein‐drug mixture were used for the secondary structure analysis. The second derivative analysis was applied to the absorbance spectra to distinguish overlapping bands without introducing any bias. This was accomplished using a second‐order Savitzky‐Golay filter with a seven‐point calculation window. The number of sub‐bands and their peak positions, obtained from this analysis, were used to extract secondary structure information through Fourier self‐deconvolution (FSD). The absorbance spectra were then modelled as a combination of Lorentzian/Gaussian curves, with the peak positions set to match the frequencies identified in the second derivative analysis. This process was repeated through multiple iterations until convergence was achieved. Finally, the area under each curve was integrated to determine the relative contribution of each sub‐band, representing the percentage of a particular secondary structure motif. The effect of potential amino acid side‐chain interference in the Amide I band for secondary structural analysis was assessed to be insignificant (Detailed in Section S15 and Figure S14, Supporting Information), and therefore any relevant subtraction methods were excluded from the analysis.

### In Vitro Preparation of aSyn Structural Species

a) Monomers

The overexpression and purification of human WT aSyn in a recombinant E‐coli coli‐based system was carried out as described previously.^[^ ^86^ ^]^ Briefly, the transformation of pT7‐7 plasmid encoding WT aSyn into the BL21(DE3) E. coli cells on an ampicillin agar plate was performed, and the plate was placed in a 37 °C incubator overnight. The next day following the transformation, a single colony was transferred to 200 ml of Luria broth (LB) medium containing ampicillin (100 µg ml^−1^; AppliChem, A0839) and incubated overnight at 180 rpm at 37 °C. The following day, the preculture was used to inoculate 6 liters of LB medium containing ampicillin (100 µg ml^−1^) with the starting absorbance at 600 nm of the big culture between 0.05 and 0.1. Upon reaching the absorbance between 0.4 to 0.6 at 600 nm, aSyn protein expression was induced by the addition of 1 mM 1‐thio‐β‐D‐galactopyranoside (AppliChem, A1008), and the cells were further incubated at 180 rpm at 37 °C for 4 to 5 h. Cells were harvested by centrifugation (Thermo Scientific Sorvall LYNX 6000 Superspeed Centrifuge) at 4000 rpm using a fix‐ed angle rotor (Thermo Scientific Fiberlite F9‐6 × 1000 LEX) for 20 min at 4 °C. The harvested pellets were stored at −20 °C until use. Cell lysis was performed by dissolving the bacterial pellet in 100 ml of buffer A (40 mM tris‐HCl, pH 7.5) containing protease inhibitors (1 mM EDTA (Sigma–Aldrich, catalogue no. 11 873 580 001) and 1 mM phenylmethylsulfonyl fluoride (PMSF; AppliChem, A0999)), followed by ultrasonication (Vibra‐Cell VCX 130, Sonics, Newtown, CT) at the following parameters: 8 min; cycle: 30 s on, 30 s off; amplitude 70%. After lysis, centrifugation at 10,000 rpm at 4 °C for 30 min was performed to collect the supernatant. This supernatant was collected in 50‐ml Falcon tubes and placed in boiling water (≈100 °C) for ≈12–15 min. This solution was subjected to another round of centrifugation at 12,000 rpm at 4 °C for 30 min. The supernatant obtained at this step was filtered through 0.45‐µm filters and injected into a sample loop connected to HiPrep Q Fast Flow 16/10 (Sigma–Aldrich, GE28‐936‐543). The supernatant was injected at 2 ml min^−1^ and eluted using buffer B (40 mM Tris‐HCl, 1 M NaCl, pH 7.5) from 0 to 70% gradient at 3 ml min^−1^. All fractions were analyzed by SDS‐PAGE, and the fractions containing pure aSyn were pooled, flash‐frozen, and stored at −20 °C until use. Fractions containing the purified aSyn were loaded on a reverse‐phase HPLC (high‐performance liquid chromatography) C4 column (PROTO 300 C4 10 µm, Higgins Analytical; buffer A, 0.1% trifluoroacetic acid (TFA) in water; buffer B, 0.1% TFA in acetonitrile), and the protein was eluted using a gradient from 35 to 45% buffer B over 40 min (15 ml min^−1^). Elution of aSyn from HPLC was analyzed using ultraperformance liquid chromatography (UPLC) and electron spray ionization‐mass spectrometry (ESI‐MS). Fractions containing highly purified aSyn were pooled, snap‐frozen, and lyophilized. Lyophilized aSyn was stored at −80 °C for later use.

b) Dopamine‐Oligomers

DA‐induced oligomers were prepared according to the method described by Mahul‐Mellier et al.^[^ ^87^ ^]^ In brief, recombinant aSyn protein was dissolved in a buffer of 20 mM Tris and 100 mM NaCl to achieve a final concentration of 140 µM (pH 7.4). The solution was then filtered using a 100 kDa filter (MERCK, MRCFOR100) to remove large particles. The filtrate was transferred into a low‐protein binding tube, and 20 equivalents of dopamine (final concentration: 2.8 mM) (Sigma–Aldrich, H8502) were added. The solution was incubated in a shaking incubator at 37 °C and 200 rpm for five days in a tube covered in aluminum foil. After incubation, the sample was centrifuged at 12000 g for 10 min at 4 °C to remove any insoluble aSyn aggregates. The resulting supernatant was loaded into a sample loop of a chromatography system and passed through a Superdex 200 Increase 10/300 GL column (GE Healthcare, 28 990 944) pre‐equilibrated with PBS. Elution was performed in 0.5 mL fractions at a flow rate of 0.4 mL min^−1^, with protein elution monitored by UV absorbance at 280 and 214 nm. The oligomer fractions of interest were collected and stored at −80 °C.

c) HNE‐Oligomers

HNE‐induced oligomers were prepared following the method outlined by Näsström et al.^[^ ^88^ ^]^ In summary, recombinant aSyn protein was dissolved in a buffer containing 20 mM Tris and 100 mM NaCl to a final concentration of 140 µM (pH 7.4). The solution was then filtered using a 100 kDa filter (MERCK, MRCFOR100) to remove large particles. The filtrate was transferred into a low‐protein binding tube, and 30 equivalents of HNE (Cayman Chem, 32 100) were added, resulting in a final concentration of 4.2 mM. This mixture was incubated at 37 °C under static conditions for 18 h. After incubation, the sample was centrifuged at 12000 g for 10 min at 4 °C to eliminate any insoluble aSyn aggregates. The supernatant was loaded into a sample loop of a chromatography system and passed through a Superdex 200 Increase 10/300 GL column (GE Healthcare, 28 990 944) pre‐equilibrated with PBS. Protein elution was carried out in 0.5 mL fractions at a 0.4 mL min^−1^ flow rate, with UV absorbance monitored at 280 and 214 nm. The SEC oligomer fractions of interest were collected and stored at −80 °C.

### Transmission Electron Microscopy

7 µL of the sample were deposited on a glow discharged (with ELMO Glow Discharge system) 200 mesh Formvar‐coated TEM grid and incubated for 120s. The grids were washed three times with MilliQ water and stained with 1% uranyl formate for 60s (Electron Microscopy Sciences, UK), freshly prepared by the BioEM facility at EPFL. The grids were air‐dried for 10 min prior to imaging using a 120 keV Tecnai Spirit BioTWIN TEM in the CIME facility at EPFL.

## Conflict of Interest

The authors declare no conflict of interest.

## Author Contributions

D.K, S.T.K, H.A.L, and H.A conceived the study. D.K. conceptualized and designed the plasmonic microarray sensors. D.K. optimized and performed the numerical simulations, cleanroom fabrication of microarray sensors, protein spotting, and bioassay experiments, infrared measurements, data analysis, and interpretation. E.M. optimized the TEM imaging protocol. E.M.and D.K. performed the TEM image analysis. S.T.K. optimized the fluorescence assay protocol. S.K.T. and D.K. performed the fluorescence assay. B.D. performed fabrication, protein spotting, and infrared measurements. H.A.L. and H.A secured the funding and resources. All authors validated the results. D.K., H.A.L., and H.A. wrote the original draft. All authors contributed toward revising and editing the manuscript.

## Supporting information



Supporting Information

Supplemental Video 1

## Data Availability

The data that support the findings of this study are available from the corresponding author upon reasonable request.
