# Supplementary material for: Nanoplasmonic Infrared Microarray Sensor Enabling Structural Protein Biomarker‐Based Drug Screening for Neurodegenerative Diseases
Source: Adv Sci (Weinh). 2025 Jul 28;12(39):e00320. doi: 10.1002/advs.202500320 (PMC12533156; doi:10.1002/advs.202500320)
Supplement: Supplementary file 1 — Supporting Information [file ADVS-12-e00320-s002.docx]

Supporting Information

Nanoplasmonic infrared microarray sensor enabling protein structural biomarker-based drug screening for neurodegenerative diseases

Deepthy Kavungal*, Enzo Morro, Senthil T Kumar, Berkay Dagli, Hilal A Lashuel* and Hatice Altug*

D. Kavungal, B.Dagli, H.Altug

^1^Bionanophotonic Systems Laboratory (BIOS), Institute of Bioengineering, École Polytechnique Fédérale de Lausanne, 1015, Switzerland.

D. Kavungal, E. Morro, S. Kumar, H. Lashuel

^2^ Laboratory of Molecular Neurobiology and Neuroproteomics (LMNN), Institute of Bioengineering, École Polytechnique Fédérale de Lausanne, 1015, Switzerland.

S. T. Kumar

^3^ National Centre for Cell Science, Pune, 411007, India.

Corresponding email address: deepthy.kavungal@epfl.ch, hilal.lashuel@epfl.ch, hatice.altug@epfl.ch

Keywords: infrared spectroscopy, metasurfaces, plasmonics, microarray, neurodegenerative diseases, high-throughput screening, alpha-synuclein

**
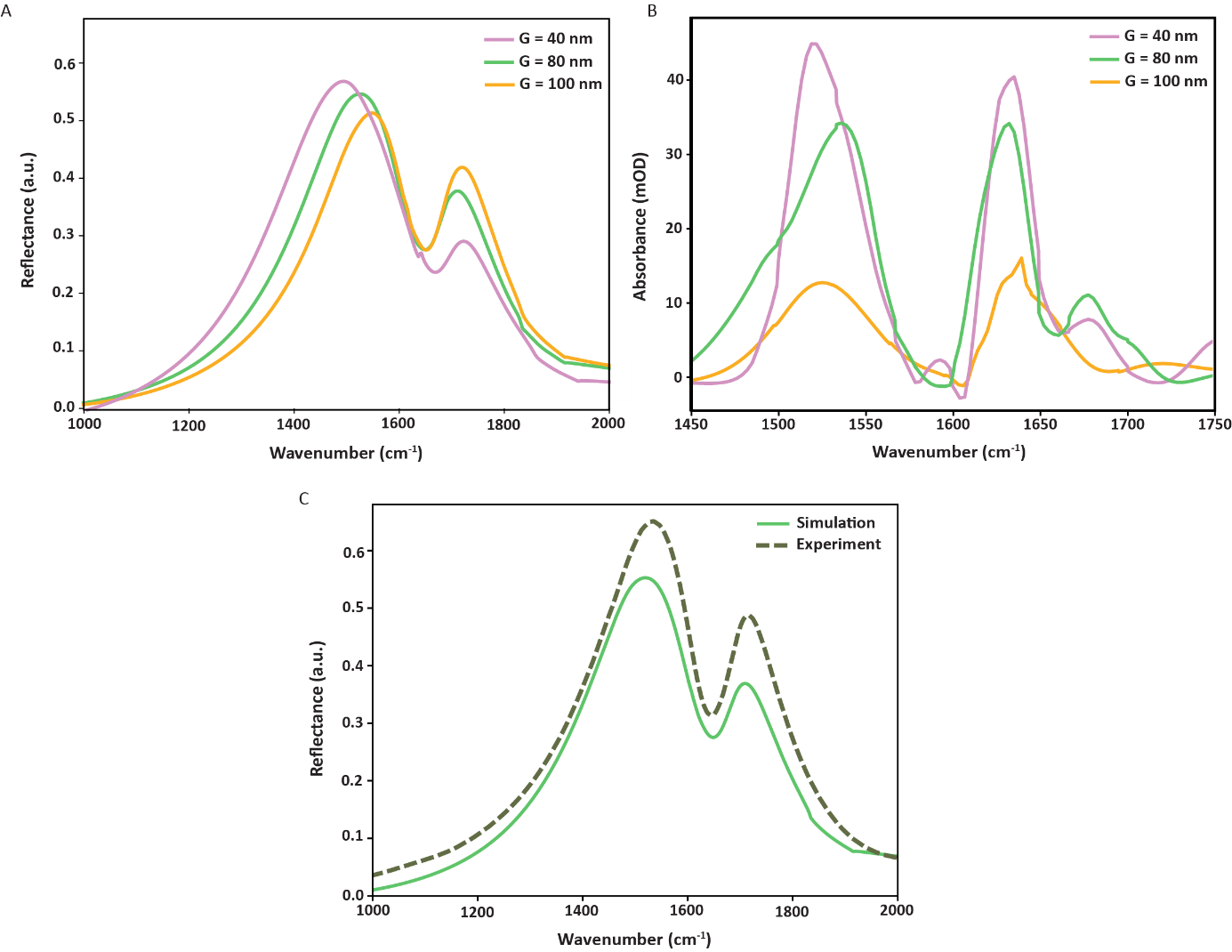
1. Optimizing the nanogaps and the comparison of the resonance response**

**Figure S1:** (A) Simulated resonance response curves of the nanostructure arrays with varying nanogaps and (B) the corresponding simulated absorbance response obtained from a 5 nm thin model layer protein on the nanostructures. (C) Resonance response curve tuned through simulation (solid green curve) and its direct comparison with the experiment reflection curve (dotted green curve) obtained from the fabricated sensor with the same parameters.

For the optimum operation of the sensor, it is crucial to match the plasmonic resonance frequency of the sensing unit with the specific absorption bands of proteins, such as the amide I and II bands (ranging from 1700 to 1500 cm⁻¹). This is achieved by adjusting the parameters of the plasmonic sensing unit, such as the length, width and height of the nanorod antenna, the periodicity and the lattice choice of the antenna arrangement and the refractive index of the surrounding medium. While fine tuning these parameters for a good spectral match, we also considered simultaneously enhancing the sensitivity of the sensor.

The GONG structure in this study was chosen to combine the strengths of the straightforward spectral tunability of nanorods through design parameters with the intense near-field enhancement achievable through the nanoscale gaps and grating order coupling. ^[44]^ Specifically, we used numerical simulations to systematically study the impact of key parameters -including length of nanorod (L), nanogap size (G), and y-periodicity (P_y_) on the spectral response and resonance tuning within the desired protein absorbance window (1500–1700 cm⁻¹).

We conducted a comparative simulation analysis of structures for varying only the gap size (G) across three values: 40 nm, 80 nm, and 100 nm at fixed nanorod length (L = 1500 nm) and y-periodicity (Py = 3200 nm). This variation alters the x-periodicity (Px = L + G), producing subtle resonance shifts. As shown in Figure S1 A, the resonance peak positions vary by a maximum of ~10-30 cm⁻¹ across the different gaps, indicating that the gap size has only a minor influence on the resonance placement, which remains within the targeted amide I band. Instead, the length and periodicity play the most impactful role in tuning the resonance peak position to the desired value. ^[89]^

On the other hand, the gap size plays a much more significant role in modulating near-field enhancement and, consequently, the absorbance signal strength. To evaluate this effect, we simulated the presence of protein on the nanostructures by placing a 5 nm thick analyte layer (optical constants (n,k) of streptavidin protein) over each geometry. The corresponding SEIRA absorbance spectra of the protein layer, shown in Figure S1 B, were calculated using the formula, A (mOD) = −1000 × log₁₀ (R_analyte/R_bare), where R_analyte is the reflectance response obtained with the protein coating and R_bare is the one with bare nanostructures (the curves shown in Figure S1 A). We can observe that the structures with 40 nm and 80 nm gaps produced significantly higher absorbance signals compared to those with a 100 nm gap size (almost 3-4 times). At the same time, there is only a ~25% increase in absorbance observed for the arrays with 40 nm gap size compared to that of the 80 nm gaps.

While 40 nm gaps offer slightly better near-field enhancement, we selected 80 nm gaps as the optimal design due to two key considerations: target protein accessibility and fabrication reliability. Our analytes in this study, aSyn monomers, oligomers, and fibrils, have a wide size range. For example, fibril size can reach up to 200 nm, and such large aggregates are less likely to access or interact efficiently within 40 nm gaps, diminishing the SEIRA signal enhancement. In contrast, 80 nm gaps ensure better accessibility and surface coverage for diverse protein species. From a fabrication standpoint, while Electron-beam lithography can achieve sub-20 nm features, producing uniform and reproducible 40 nm gaps across large-area microarrays is difficult to optimize, time-consuming and prone to variability. In comparison, 80 nm gaps offer better reproducibility in fabrication.

The chosen parameters from the simulation procedure are then used in the fabrication step. Figure S1 C shows the comparison between the simulated reflectance spectra (solid curve) and the measured response from the fabricated sensor (dotted curve). The results confirm that the peak positions of the resonance curves of both simulation and experiment match very well. The slight difference in the intensity is attributed to the larger array (500 x 500 µm^2^) used in the experiment in the case of the 96-microwell microarray sensor.

Detailed procedure for the numerical simulation of the plasmonic metasurface sensing unit is provided below:

A finite integration Maxwell solver (CST Studio 2018) is employed for the numerical design and analysis of the far-field and near-field characteristics of the nanostructures. The simulation setup includes a unit cell comprising a gold (Au) nanorod, 100 nm in height, deposited on a 5 nm chromium (Cr) adhesion layer, all placed on a calcium difluoride (CaF₂) substrate. A water layer is added above the antennas. The optical constants for Au, Cr, CaF₂, and water are taken from Olmon et al., Rakić et al., Li, and Hale et al., respectively.^[90–93]^ The structures are modeled using a tetrahedral mesh with a 40 nm element size and simulated under periodic boundary conditions. The incident plane wave is configured at an average inclination angle of 16.7°, corresponding to the angular range (9.8° to 23.6°) of the Cassegrain objective used in the FTIR measurements. The far-field optical response is obtained by averaging the spectra from both transverse magnetic (TM) and transverse electric (TE) excitation modes, with TM polarized perpendicular to the nanorod’s long axis and TE perpendicular to its short axis.

1. **AFM and SEM characterization of the Au nanorods and SU-8 microwells**


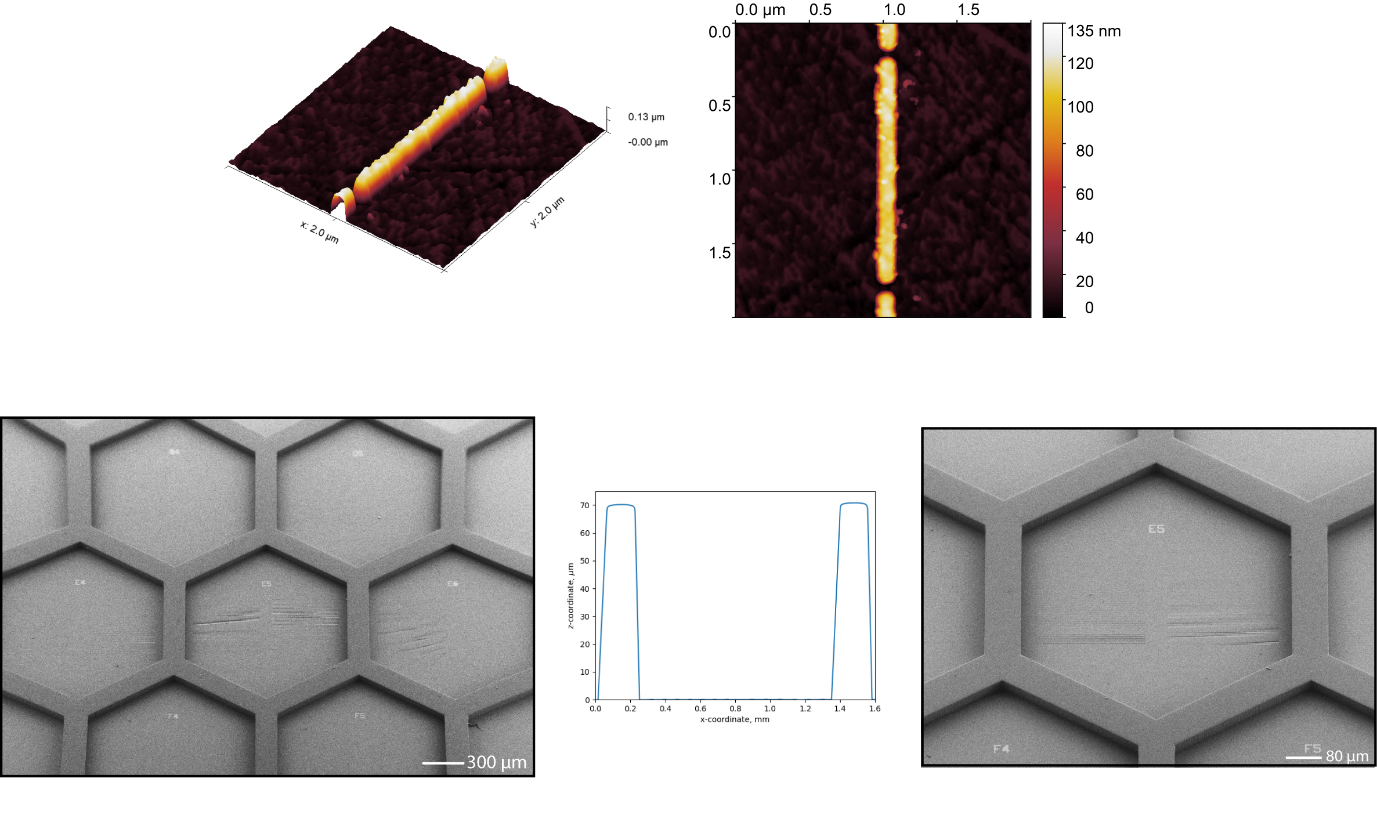


**Figure S2**: AFM characterization of the individual nanorods and the SEM images of the SU-8 microwells with the height profile of the honeycomb walls.


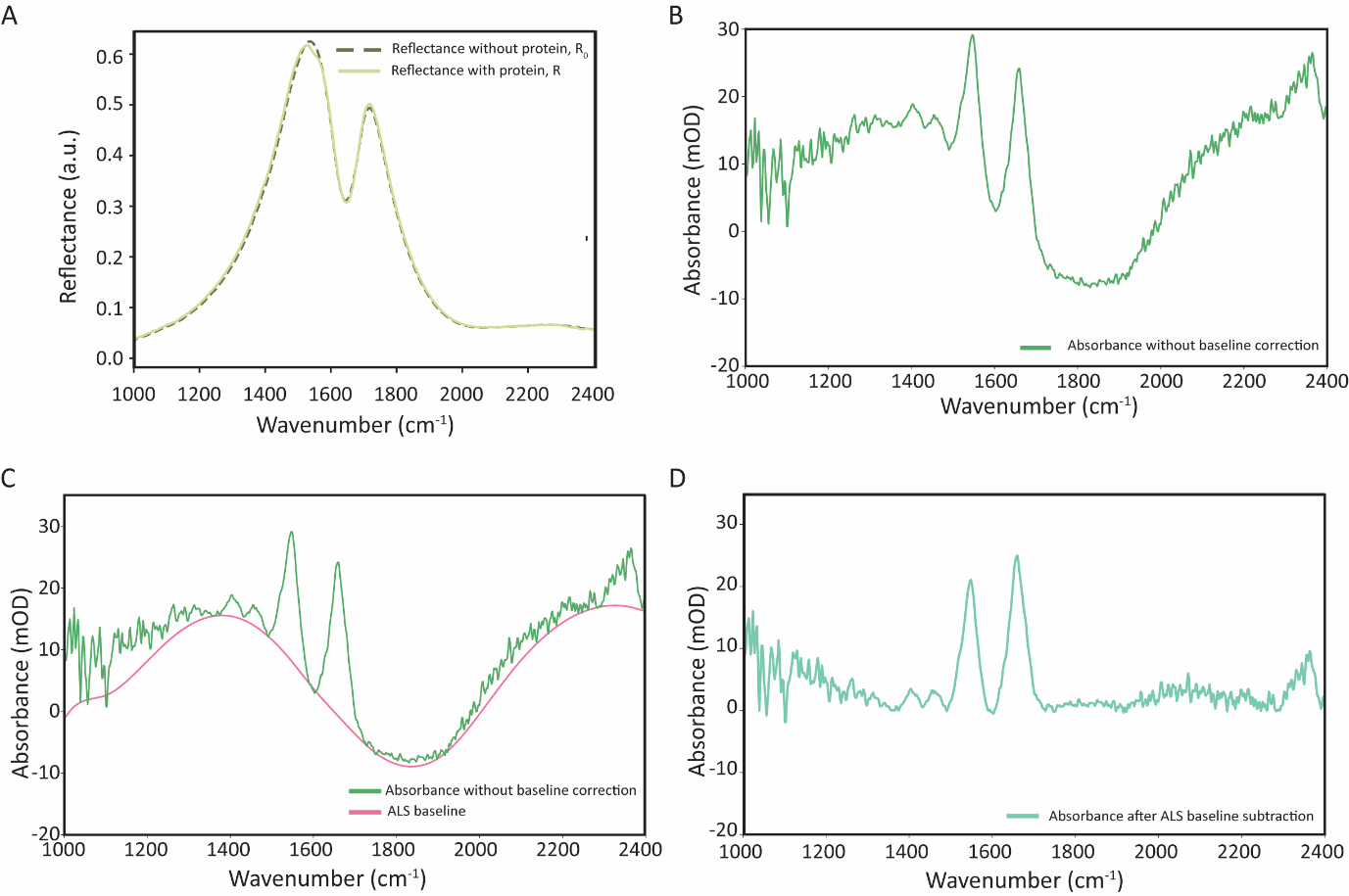
**3. Asymmetric Least Square (ALS) baseline correction procedure**

**Figure S3:** Asymmetric Least Squares (ALS) baseline correction. **(**A) Reflectance of the sensing units without (R_0_) and with the protein (R). (B) Raw absorbance calculated using the formula -1000*log_10_ (R/ R_0_). (C) Baseline fit calculated using the ALS algorithm (pink curve) overlapping with the raw absorbance spectra (green curve). (D) Final absorbance obtained after subtracting the ALS baseline.

In this study, we employed the Asymmetric Least Squares (ALS) smoothing-based baseline correction method by Eilers et al. ^[85]^. Figure S3 A shows the reflectance of the sensing units with resonance around 1600 cm^-1^ in wet conditions (legend ‘Reflectance without protein’, R_0_) and the reflectance collected after the BSA protein bound to the thiols on the sensor surface (legend ‘Reflectance with protein’, R). The small dips in the spectrum R around 1500-1700 cm^-1^ and the spectral shift indicate successful protein capture. Calculation of the differential absorbance (mOD) from the protein is then calculated using the formula -1000*log_10_(R/R_0_) to obtain the spectra in Figure S3 B, clearly showing the asymmetric line profile typically observed in SEIRA measurements. The baseline fit is generated using the ALS algorithm with the parameters mentioned in the Experimental Section (Figure S3 C, pink curve). The ALS baseline is subtracted from the raw differential absorbance to get the final absorbance spectra, as shown in Figure S3 D.

1. **Stability of the thiol functionalized SEIRA plasmonic microarray sensor**

We evaluated the stability of the sensor in terms of its capability to stay intact after the plasmonic units are functionalized with the activated ester thiols and stored at 4°C under ambient conditions. We quantify this by measuring the amount of binding when the 96- microwell sensors are spotted with the BSA protein (specifically, the peak absorbance value of BSA at 1650 cm^-1^) after different durations of storage. The plot in Figure S4 gives an overview of the obtained results.


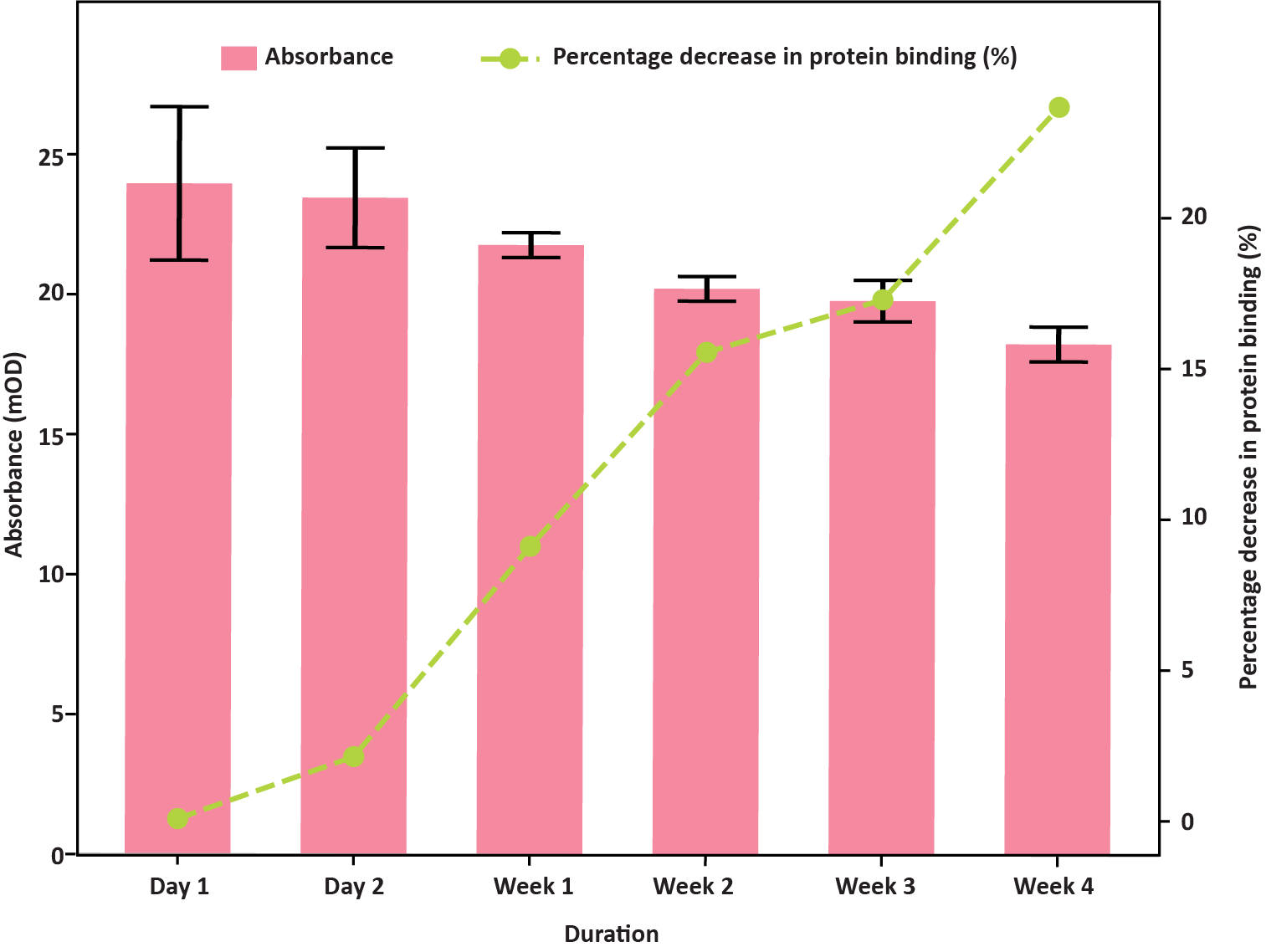
The stability analysis was done for a maximum of 4 weeks, and separate 96-microwell microarray sensors were used for each measurement at a specific time period. From the bar plot, despite the high hydrolysis nature of the activated thiols, the protein binding is relatively stable. There is a decreasing trend of protein binding as the duration increases. To better quantify it, the percentage decrease of protein binding is also plotted as a trend line plot (green dotted curve). Within the first week, less than 10% of binding is reduced and increases to about ~22% by week 4. The gradual decrease in binding could be attributed to the gradual hydrolysis of the activated NHS-ester thiols due to its constant exposure to moisture in the air while stored in a standard laboratory refrigerator.

**Figure S4**: Bar plot showing the amount of protein binding and a trend line plot showing the percentage decrease of the protein binding over the duration of the storage.

The results suggest that the microarray sensor has good stability, and the chip surface remains active and effective for biomolecular interaction and protein binding capacity even after extended storage in a non-ideal storage environment. Importantly, storage under optimized and inert conditions like a dry N₂ atmosphere or vacuum sealing of the chip after thiol functionalization can further enhance the shelf-life, stability and performance of the sensor.

1. **Chemical composition of the 9 drugs used in the fluorescence assay and the plasmonics SEIRA microarray measurements**


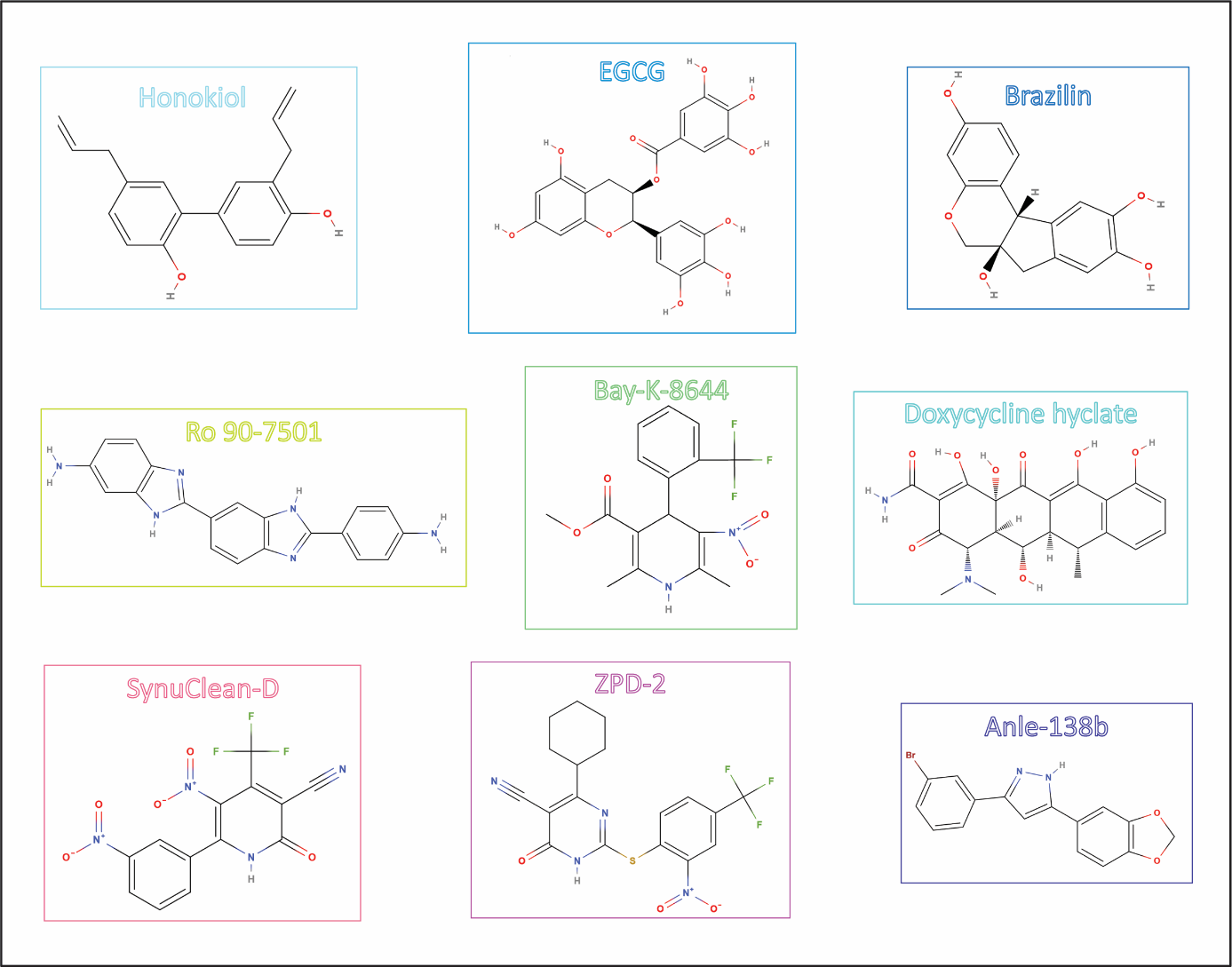


**Figure S5**: Chemical formula of the 9 drugs used in the fluorescence assay and the plasmonics SEIRA microarray measurements.

1. **Negative control for fluorescence assay experiments**

ThT fluorescence assay was performed to assess the effect of drugs and their interaction with the ThT dye in the absence of the aSyn protein. The results from Figure S6 A and B show that except for the drug compound Anle, all other drug compounds exhibit some quenching effect, as seen from their corresponding fluorescence intensities being much lower than the blank sample that contains only ThT dye and buffer (PBS 1x).


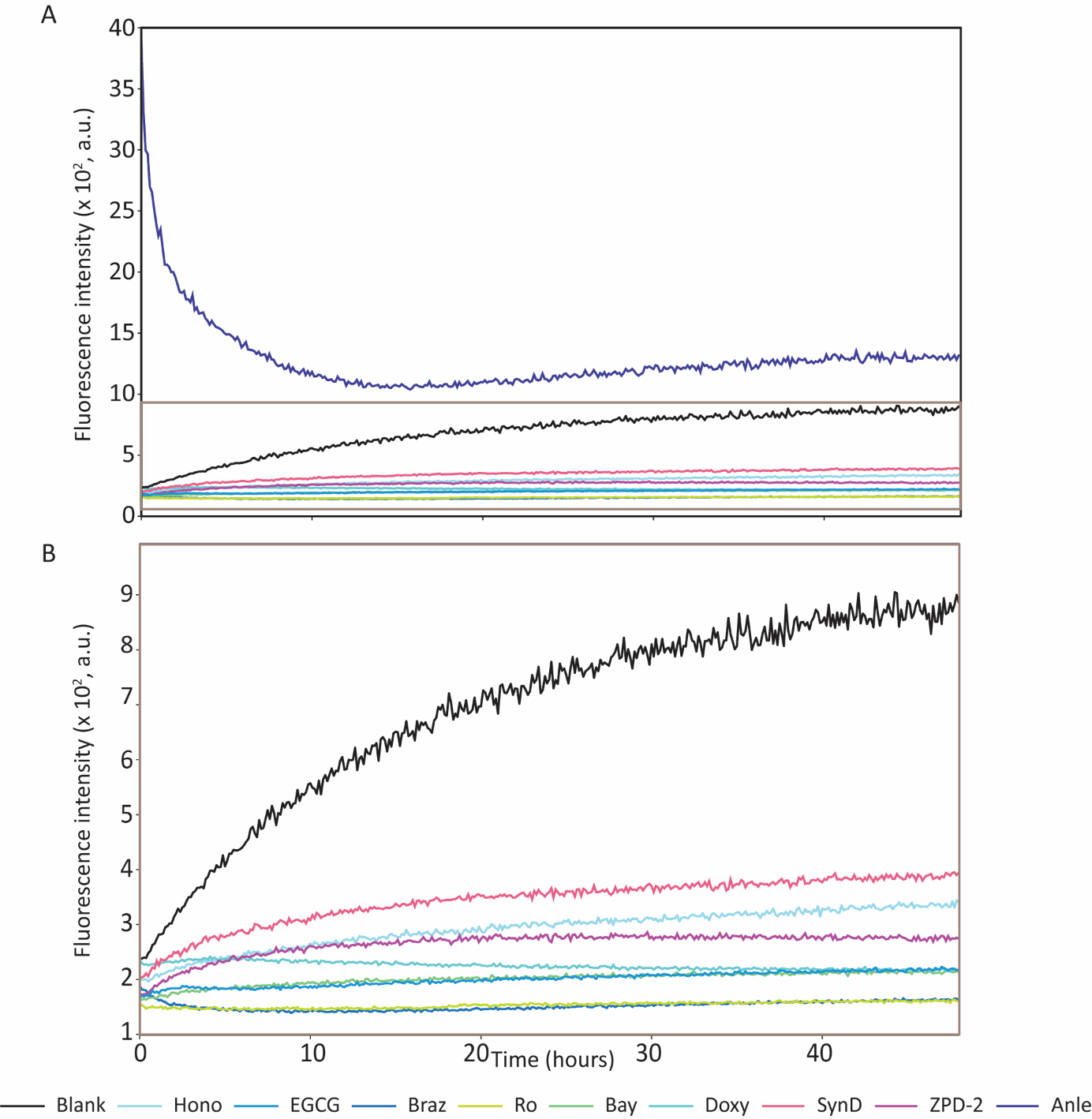


**Figure S6**: (A) ThT fluorescence assay results from the negative control experiments using only ThT dye and drugs without the aSyn protein. (B) Zoomed-in plots without Anle for better visualization of the responses from other drugs.

1. **Fourier self-deconvolution (FSD) and curve fitting for quantitative structural analysis.**


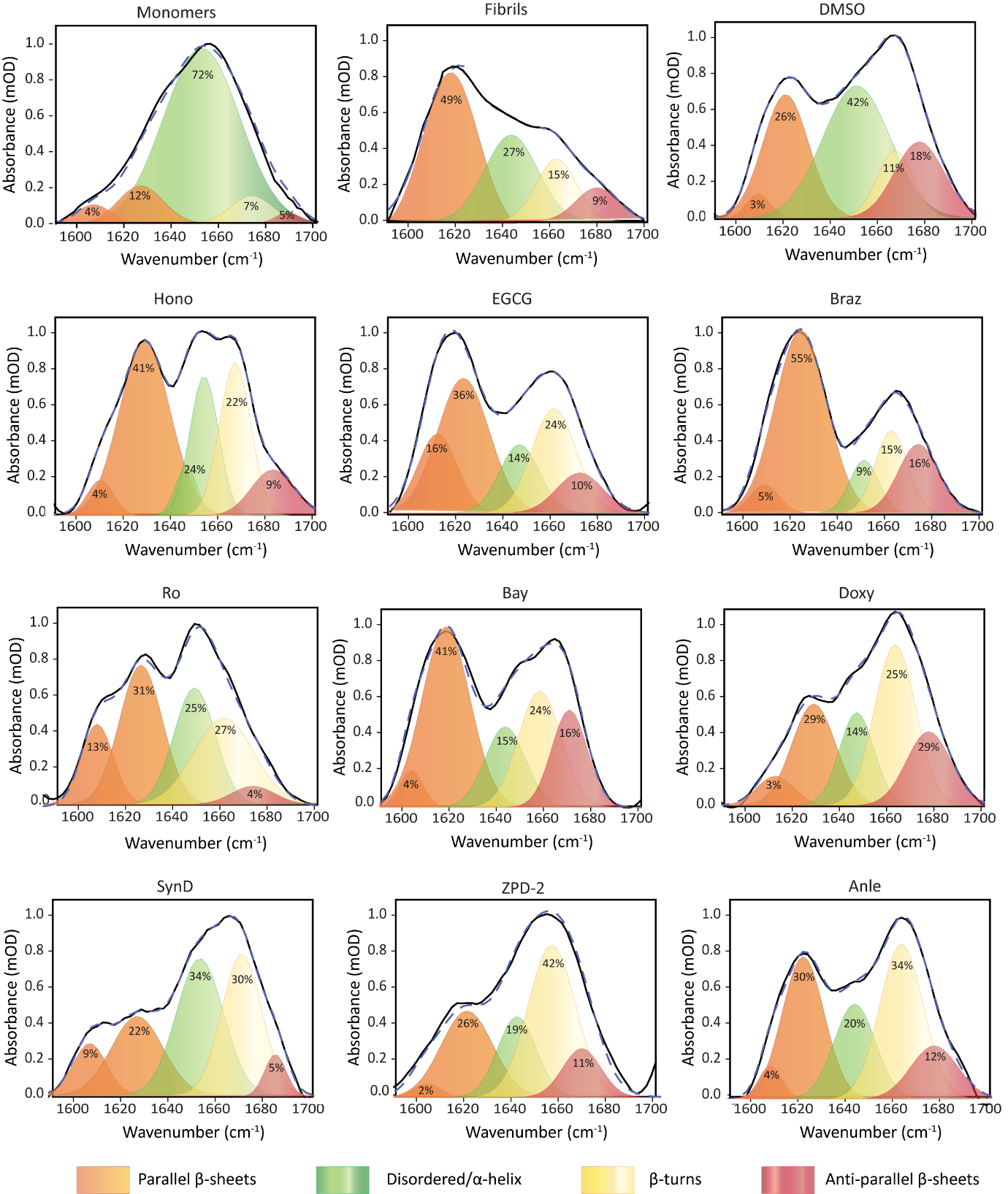


**Figure S7**: Quantitative structure analysis of drug-incubated protein samples with percentages of distinct secondary structural motifs in the mixture obtained using Fourier Self-deconvolution (FSD) and curve-fitting.

1. **Negative control experiment to analyze the interaction between the drug compounds and the plasmonic substrate**

To rule out any potential drug-substrate interaction during the SEIRA measurement phase that could possibly influence the structural response measured, we performed negative control experiments where individual drugs were spotted on the 96-microwell plasmonic microarray chip at their assay concentrations (500 µM) in the absence of any protein. The chip was then incubated, washed, and measured under identical conditions to the main experiments. As shown in Figure S8, the measured absorbance spectra of these drug-only samples are comparable to those obtained from unspotted (blank) microwells, indicating negligible or no binding of the drugs to the gold-thiol surface under our experimental conditions.


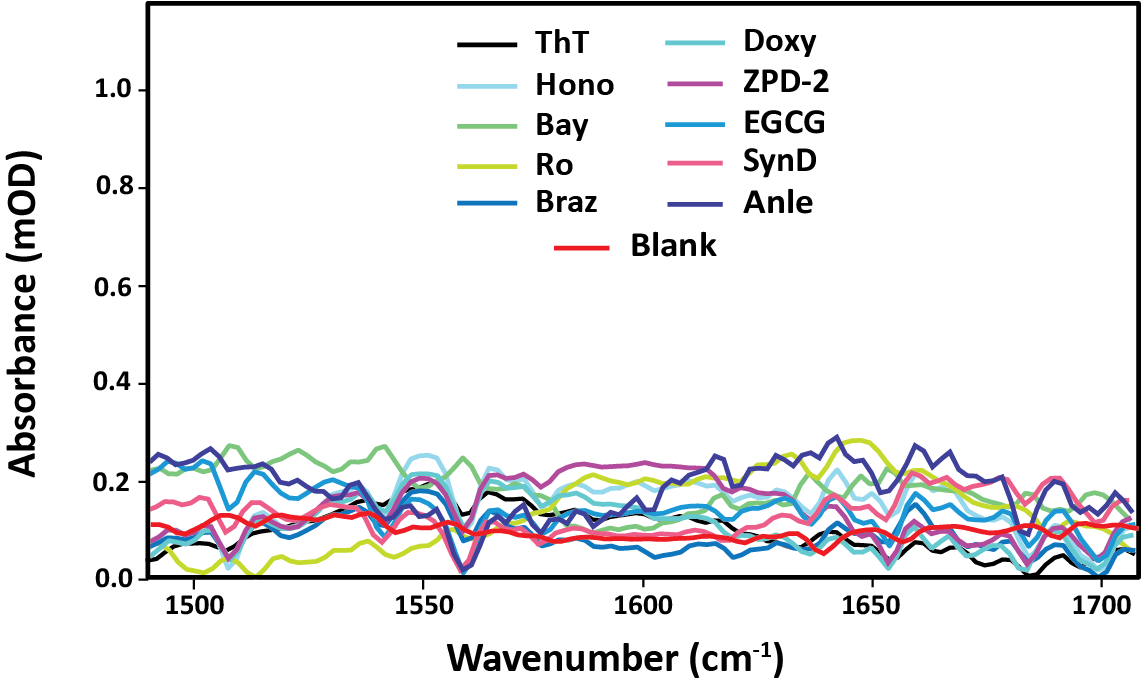


**Figure S8**: Absorbance spectra of the drugs-only sample incubated on the 96-microwell SEIRA sensor.

These new results indicate that the surface chemistry of our SEIRA microarray chip does not introduce spectral background or alter protein-drug interactions post-incubation. Thus, our methodology ensures that the measured secondary structure profiles reflect the actual aggregation outcomes in free solution, without any substrate-induced bias.

1. **Structural integrity of aSyn protein on the sensor surface**

We performed extended time-course experiments to observe any change in the secondary structure profile of samples when the same microwells were re-measured hours after the initial scan. Figure S9 shows the corresponding experimental data. We retrieved the Amide I band absorbance of 50 µM aSyn monomers spotted on the same microwell of 96-microwell plasmonic SEIRA sensor at 3 different timepoints: 1) Time 0 – at the start of the in situ experiment, 2) Time + 2hrs – after 2 hrs of starting the measurement and 3) Time > 24 hrs – after extensive measurement on the first day (over 3 to 4 hours), the chip is stored at 4°C overnight for more than 24 hours and re-measured again the next day (Measurement parameters are the same as detailed in the Experimental Section). Figure S9 A and S9 B show the Amide I bands and the corresponding quantitative structural response, respectively. From the data, we can infer that the band response and the percentage contributions of different secondary structures do not show significant differences over these different time points. This suggests that our measurement process does not induce any structural alterations due to resonance-induced localized heating or optical effects from the plasmonic substrate within the experimental timeframe and workflow of this study.


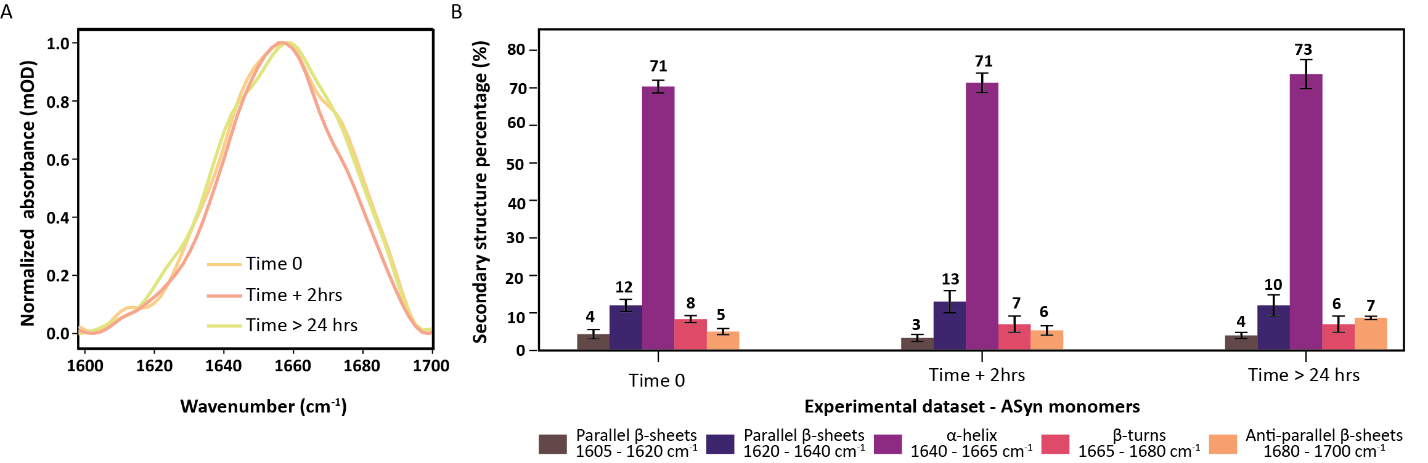


**Figure S9:** (A) Amide I band response and (B) the corresponding quantitative secondary structure percentages of aSyn monomers spotted on the same microwell measured at different timepoints.

1.
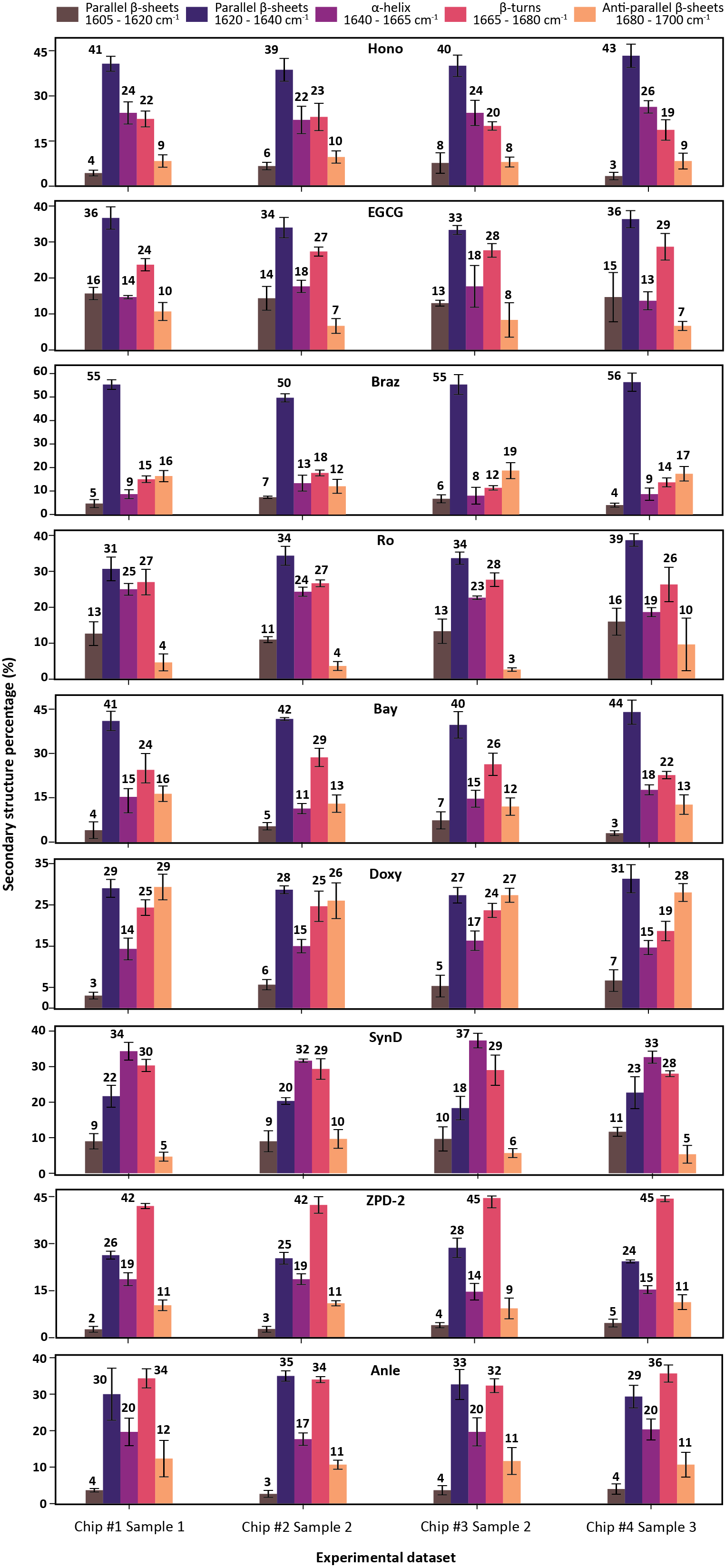
**Reproducibility study of the quantitative structural analysis of aSyn-drug mixture**

**Figure S10**: Quantitative secondary structure analysis of aSyn monomers treated with different drug candidates across multiple chips and samples. Bar plots represent the percentage contribution of various secondary structure motifs - parallel β-sheets (1605 - 1620 cm⁻¹ and 1620 - 1640 cm⁻¹), α-helix (1640 - 1650 cm⁻¹), β-turns (1660 - 1685 cm⁻¹), and antiparallel β-sheets (1680 - 1700 cm⁻¹), for each of the 9 drugs measured on four independent chips (Chip #1 to Chip #4) and three independently prepared sample sets (Sample 1, 2 and 3) as different replicates. The values show the average of the results obtained plotted with the error bars.

Good reproducibility is a critical criterion for any sensor intended for high-throughput drug screening applications. Therefore, we performed a reproducibility study involving multiple independent experimental runs, using different sensor chips and independently prepared sample sets.

We conducted four separate experiments using four different 96-microwell plasmonic SEIRA sensor chips (Chip #1 to Chip #4) and three independently prepared sample sets (Sample 1, 2, and 3) of aSyn monomers treated with the same nine drugs presented in the study. Each sample was prepared at a protein-to-drug ratio of 1:10 and incubated under identical conditions (48 hours at 37 °C and 600 rpm) to minimize experimental variability. To directly assess consistency across microarray chips, we included Sample 2 on both Chip #2 and Chip #3. Each drug-treated sample was spotted in triplicate across the microwells to evaluate the consistency within the same microarray chip. The dataset shown for Chip #1 Sample 1 is the same as presented in Figures 3, 4 and 5 of the main manuscript and reproduced here for comparative purposes.

We analyzed the secondary structure compositions using the Fourier self-deconvolution (FSD) protocol, as described in the main sections (Figure 5 in the Results section and the Experimental Section under Secondary structure analysis) from the Amide I band retrieved for each of the replicates across different experiments. The results from these reproducibility experiments are summarized in Figure S10, which presents the quantitative secondary structure contributions for each drug, i.e., the percentages of parallel β-sheets (1605 - 1620 cm⁻¹ and 1620 - 1640 cm⁻¹), α-helices (1640 - 1650 cm⁻¹), β-turns (1660 - 1685 cm⁻¹), and antiparallel β-sheets (1680 - 1700 cm⁻¹). The values plotted are the average of three replicates, shown with error bars representing standard deviation.

We observed consistent reproducibility with minor variations across microarray chips and sample sets. For example, Braz consistently exhibited a strong parallel β-sheets content (4 - 7% and 50 - 56%) across all samples and chips. Similar consistent aggregation patterns were observed for EGCG (13 - 16%, 33 - 36%), Hono (3 - 8%, 39 - 43%), Ro (11 - 16%, 31 - 39%), and Bay (3 - 7%, 40 - 44%), all showing reproducible and predominant parallel β-sheets signatures. Doxy-treated samples maintained dominant parallel β-sheets (3 - 7%, 27 - 31%) and antiparallel β-sheet (26 - 29%) contributions across replicates. ZPD-2-treated samples showed consistent β-turns dominance (42 - 45%) with lower parallel β-sheets contributions (2 - 5%, 24 - 28%), and Anle-treated samples repeatedly demonstrated a nearly balanced composition between parallel β-sheets (3 - 4%, 29 - 35%) and β-turns (32 - 36%).

We equally observed some minor variations throughout the individual datasets, which are expected due to biological and experimental variability. For example, one Bay-treated dataset (Chip #2 Sample 2) showed a slightly higher α-helix contribution than others, and Ro-treated samples showed a higher antiparallel β-sheets content in Chip #4 Sample 3, along with slightly larger error bars. These variations are likely due to subtle differences in aggregation kinetics, drug-protein interactions or errors induced during sample preparation/incubation and indirectly highlight our platform’s sensitivity to minor structural changes. Importantly, across all drugs, we consistently captured distinct structural fingerprints, confirming the microarray sensor’s ability to detect subtle but reproducible drug-induced aggregation behaviours.

These results demonstrate both the excellent repeatability of the measurements when experimental protocols are carefully followed and the importance of performing multiple independent experiments to capture the natural variability and achieve the highest degree of accuracy possible. They also underline the need for meticulous validation in any high-throughput drug screening platform.

1.
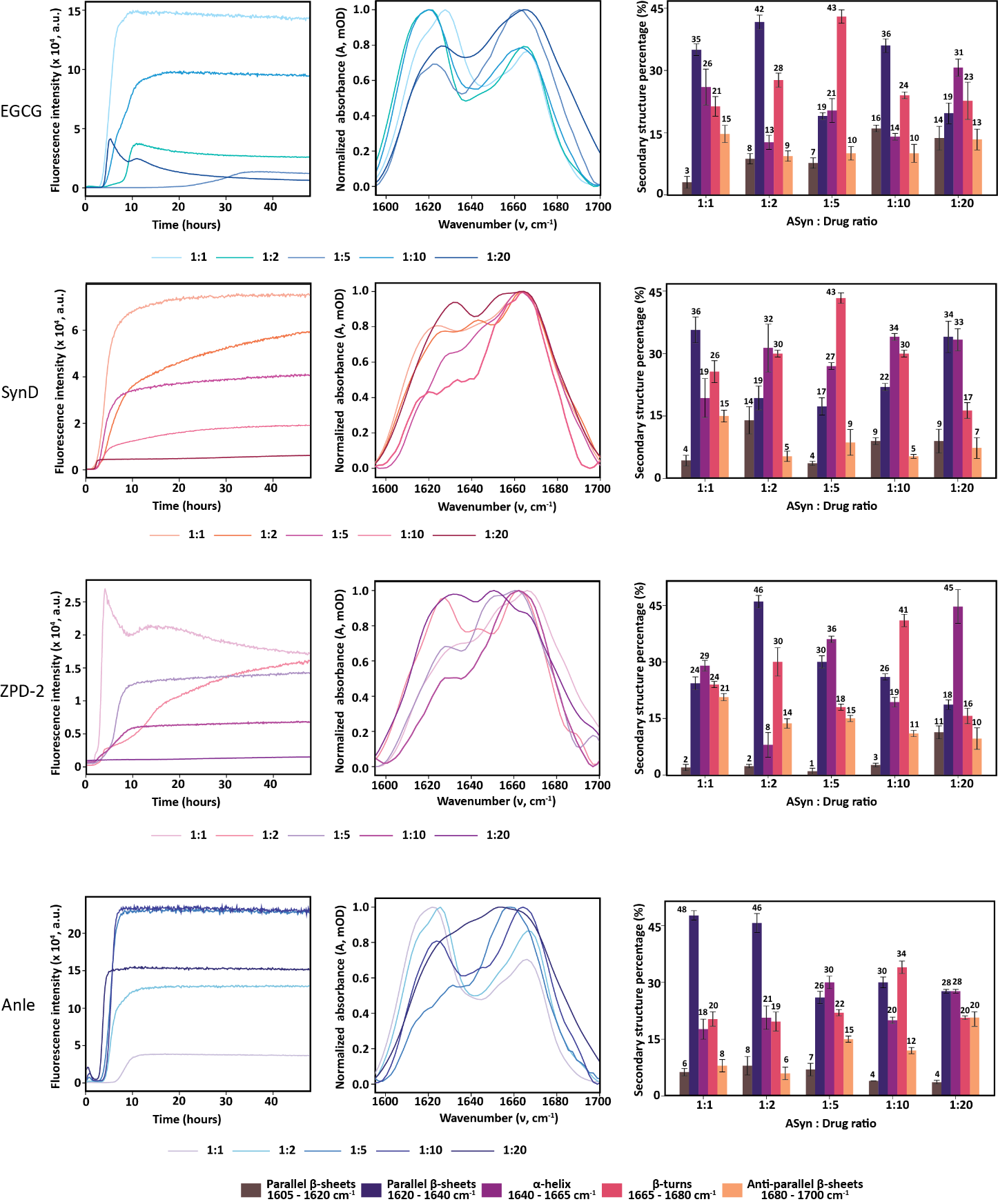
**Dose-dependent structural response analysis**

**Figure S11**: Dose-dependent response analysis of aSyn in the presence of four different small molecule drugs (EGCG, SynD, ZPD-2 and Anle) using ThT fluorescence assay (left), SEIRA spectroscopy (center), and quantitative structural deconvolution from the Amide I band (right).

A dose-dependent response (pharmacodynamic) analysis of the drugs with the potential target allows for a better understanding of the therapeutic effect of the drug and simultaneously reveals the relationship between drug concentration and its structural impact on the target structural protein biomarker.

Therefore, we conducted a detailed drug concentration-response analysis for four small molecule drug candidates - EGCG, SynD, ZPD-2, and Anle, which are among the most extensively studied and promising compounds for the prevention and/or disaggregation of aSyn aggregates. For each drug, we used five different aSyn: drug molar ratios - 1:1, 1:2, 1:5, 1:10, and 1:20, with 1:10, already included in the main results (Figures 3, 4, and 5), and repeated here for comparative purposes.

The incubation protocol followed the same conditions as the main experiments: 50 µM aSyn monomer mixed with the drug at the respective concentrations (50 µM, 100 µM, 250 µM, 500 µM, and 1 mM) and ThT dye (50 µM), incubated for 48 hours at 37 °C and 600 rpm. The samples were then monitored with real-time fluorescence measurements and spotted onto a 96-well SEIRA microarray sensor chip after drug incubation and measured using the same in-situ microfluidic setup after room temperature incubation for 2 hours and washing, as detailed in the Experimental Section.

Figure S11 presents the results of this analysis. The leftmost plots show ThT fluorescence assay data for each drug across the five concentration ratios. The center plots display the normalized Amide I spectra obtained from SEIRA measurements using the microarray chip. The rightmost bar plots show the quantitative structural analysis, derived from the Amide I spectra using Fourier self-deconvolution (FSD), indicating the percentage contributions of parallel β-sheets, α-helices, β-turns, and antiparallel β-sheets. All of the ThT and SEIRA data represent the average of three replicate measurements, and the structural bar plots include error bars indicating variability across replicates.

Overall, the results demonstrate that each drug concentration ratio leads to distinct Amide I spectral signatures and, therefore, unique secondary structure compositions, even for the same compound. For EGCG, the Amide I spectra at 1:1, 1:2, and 1:10 ratios display dominant peaks in the parallel β-sheets region, corresponding to the highest calculated β-sheets contents: ~38%, ~50%, and ~52%, respectively. The 1:2 and 1:10 spectra appear very similar, even though their ThT curves differ in intensity, highlighting a discrepancy between fluorescence response and actual structural composition. In contrast, the 1:5 and 1:20 ratios show weaker but still predominant β-sheet features (~26% and ~33%, respectively), reflected by off-peak shoulders in the Amide I band. For SynD, the ThT signal decreases with increasing drug concentration, suggesting possible inhibition. However, the SEIRA results indicate otherwise: all ratios show prominent peaks in the parallel β-sheets region, with the 1:20 ratio exhibiting the highest parallel β-sheets content (~43%). The lowest β-sheets content appears at the 1:5 ratio with ~31% parallel β-sheets and ~9% antiparallel β-sheets. Similarly, all tested ratios of ZPD-2 and Anle also resulted in final products rich in β-sheets structures. For ZPD-2, the highest β-sheets content was observed at 1:2 (~48% parallel, ~14% antiparallel), while the 1:10 ratio exhibited the highest β-turns content (~41%), and the 1:20 ratio showed a modest increase in α-helix content (~45%), still lower than that of pure monomers (~72%). For Anle, the 1:1 and 1:2 ratios showed strong conversion to parallel β-sheets (~54%), with a gradual decrease at higher concentrations, yet maintaining predominance (~33%, ~34%, and ~32% for 1:5, 1:10, and 1:20, respectively). Interestingly, the ZPD-2 1:1 ratio and Anle 1:20 ratio showed a high antiparallel β-sheets contribution (~21% and ~20%, respectively), suggesting possible structural rearrangement or off-pathway aggregation at high concentrations.

Importantly, none of the drug ratios across all four compounds successfully prevented β-sheets-rich aggregate formation, emphasizing the challenge of fully inhibiting aSyn aggregation. Moreover, although ThT fluorescence profiles vary significantly with concentration, they do not reliably correlate with the actual secondary structure content, as shown by SEIRA and the corresponding quantitative structural analysis. This discrepancy underlines the limitations of ThT assays, possibly due to quenching and non-specific interactions at high drug concentrations.

Therefore, this dose-dependent study provides valuable insights into the structural impact of varying drug concentrations on aSyn aggregation. While high drug concentrations can sometimes lead to non-linear effects or off-target interactions, our results indicate that SEIRA provides a more accurate and quantitative assessment of drug-induced structural changes than ThT fluorescence alone. This experiment strongly supports the value of SEIRA-based secondary structure analysis in pharmacodynamic evaluations and illustrates the platform’s utility for high-throughput, concentration-dependent drug screening in NDDs research.

1. **ThT fluorescence assay of DA-induced and HNE-induced oligomers**

The fluorescence assay was carried out by mixing ThT dye in equal proportion to the molar concentrations of HNE- and DA-oligomers with an additional blank sample containing the same amount of ThT dye and buffer. The fluorescence values were measured using parameters used in this study, detailed in the Experimental Section and plotted as shown in Figure S12. We can observe no significant increase in the signals for both the oligomer samples compared to the blank, especially for HNE-induced oligomers, which have a β-sheets presence as observed in SEIRA experiments.


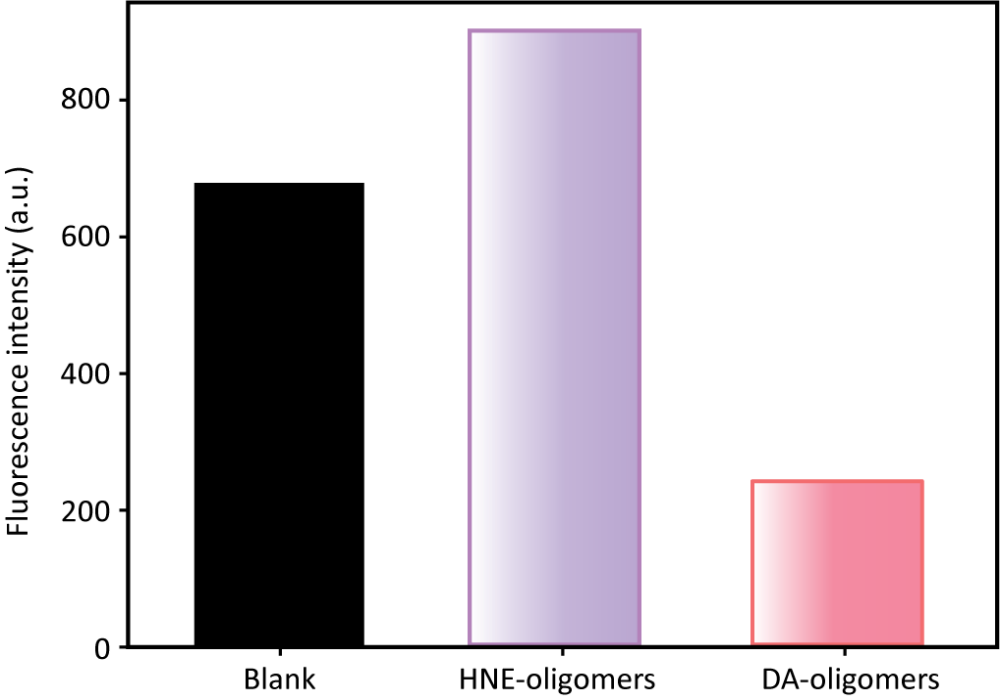


**Figure S12:** Fluorescence intensity of the HNE-oligomers and Dopamine-induced oligomers with the blank signal.

1. **
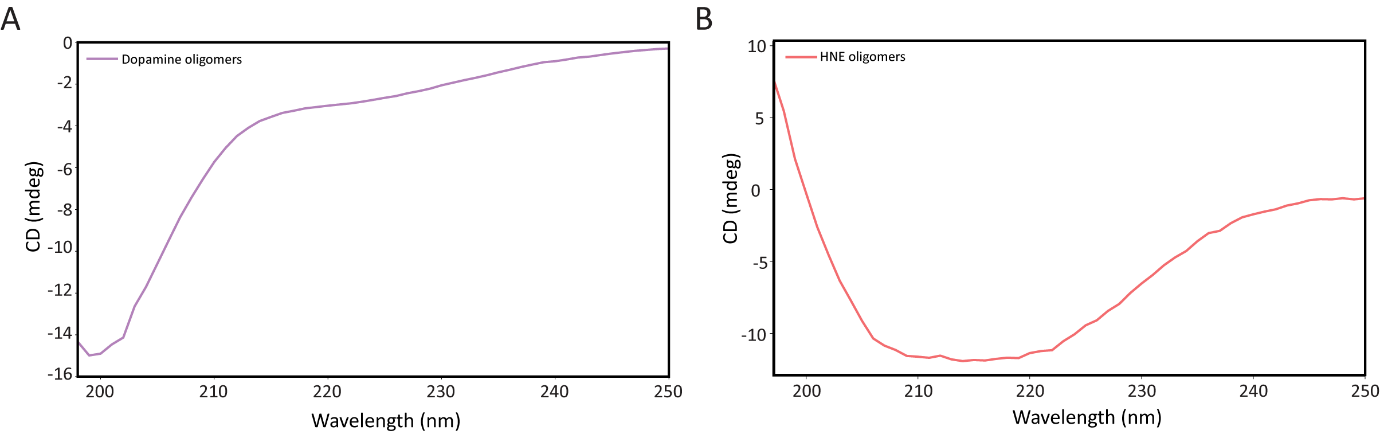
CD spectroscopy of DA-oligomers and HNE-oligomers**

**Figure S13**: CD spectra of the (A) DA-oligomers and (B) HNE-oligomers.

CD spectroscopy was performed on the final oligomeric species obtained in the drug-aSyn incubation experiments performed in section 2.4 in the main manuscript and presented in Figure S13. The CD spectra of DA-oligomers (Figure S13 A) show a minimum peak at 198 nm, indicating that the majority of the secondary structure is in the disordered form, whereas the spectra of HNE-oligomers (Figure S13 B) present a broader negative peak around 210-220 nm, pointing to the predominant presence of β-sheets.

1. **Comparison of plasmonic microarray sensor with the existing technologies**

| **Technique** | **Well-plate/microarray** | **Volume range** | **Conc. Range** | **Dry/In-situ** | **Plate/chip dimensions (mm)** | **Well dimensions (mm)** | **Qualitative/Quantitative** | **Reference** |
| --- | --- | --- | --- | --- | --- | --- | --- | --- |
| DLS | Well-plate | 50-360 µL | 0.2-10 mg/mL | In-situ | 127.8x85.5x14.2 (LxWxH) | 48 -  (d x h)  11.05 x 17.4  96 -  6.35 x 11.3 384 -  3.63 x 11.43 | Qualitative | ^[94]^ |
| ThT | Well-plate | 50-360 µL | 2.89 µg/mL-7.23 mg/mL | In-situ | 127.8x85.5x14.2 (LxWxH) | 48 -  (d x h)  11.05 x 17.4  96 -  6.35 x 11.3 384 -  3.63 x 11.43 | Qualitative | ^[95]^ |
| CD | Well-plate | 50-360 µL | 2-10 mg/mL | In-situ | 127.8x85.5x14.2 (LxWxH) | 48 -  (d x h)  11.05 x 17.4  96 -  6.35 x 11.3 384 -  3.63 x 11.43 | Semi-quantitative | ^[96]^ |
| IR (Bruker HTS-XT) | Well-plate | 1-20 µL | 0.5-3 mg/mL | Dry | Similar to standard well-plates (exact dimensions not disclosed) | Similar to standard well-plates (exact dimensions not disclosed) | Quantitative | ^[97]^ |
| Plasmonic SEIRA Microarray | Microarray | 2-270 nL | 100 pg/mL - 10 mg/mL | In-situ | 30 x 0.5  (D x H) | 48 - 2 x 0.07 (d x h) 96 -  1.38 x 0.07 384 -  0.69 x 0.07 | Quantitative | This work |

D = chip diameter, L= plate length, W= plate width, H = plate height, d= well diameter, h= well depth

**Table S1**: Comparison of plasmonic microarray sensor with the existing multiwell technologies in relevant performance metrics.

Given the unique capabilities of our plasmonic microarray sensor, we provide a performance comparison table with existing techniques used for secondary structure analysis. We should note that a direct comparison with other “microarray sensors” is challenging because existing techniques are only available in traditional “multiwell” plate format, which is restricted to large sample volumes (µL) and high concentrations. Instead, our work introduces a compact chip-based microarray sensor realized by precise micro/nanofabrication methods for performing quantitative secondary structure analysis of proteins from extremely small volumes (nL) and low analyte concentrations, thanks to the intrinsic signal enhancements provided by the SEIRA principle and the microwell design.

Therefore, Table S1 present a comparative table which includes key parameters that are relevant within the scope of our research focus.

1. **Effect of amino acid side-chain absorbance in the quantitative secondary structure analysis using the Amide I band**

In the literature, it is well recognized that the amide I band (1600 – 1700 cm⁻¹) primarily arises from backbone C=O stretching vibrations. A number of studies have investigated whether certain side chains, particularly aromatic residues like tyrosine (Tyr) and tryptophan (Trp), may exhibit weak absorptions in this region. Such potential interference is found to be minimal, thus reaffirming that the Amide I band predominantly depends on the nature of secondary structure present. ^[31,98,99]^

To validate this in the context of our study, we performed a control experiment using tyrosine as a representative amino acid residue. Given that human full-length aSyn monomers contain four tyrosine residues per molecule, we used 200 µM L-tyrosine to approximate the molar concentration of side chains found in 50 µM aSyn monomer samples. We incubated the thiolated 96-microwell plasmonic SEIRA chip with pure 200 µM L-tyrosine under identical conditions used in the main experiments (room temperature incubation for 2 hours, followed by washing and then integration with microfluidics for in situ measurements). The absorbance spectra were recorded under the same SEIRA measurement conditions as our main experiments (128 scans, ~1-minute acquisition, 4 cm⁻¹ resolution). The resulting spectra, presented in Figure S14 A, reveal a weak signal near 1620 cm⁻¹ - attributed to tyrosine C-C and C-H bonds, but with extremely low intensity, significant noise and below our Limit of Detection (LoD) absorbance strength ( ~1 mOD), consistently observed across 3 different sensor arrays.


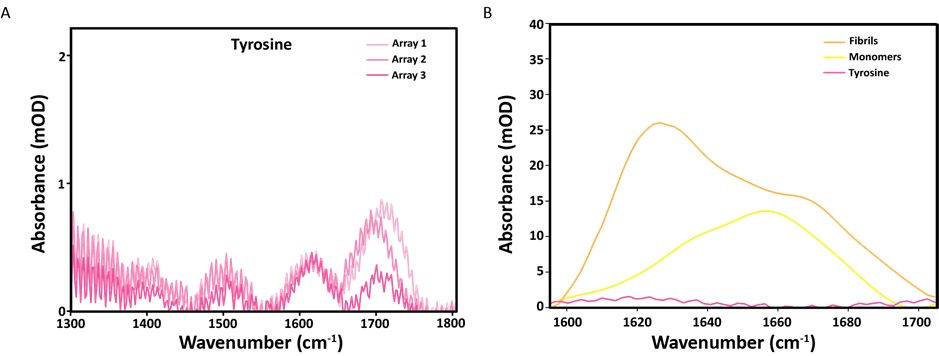


**Figure S14: (A)** SEIRA absorbance spectra of the L-Tyrosine amino acid obtained from three different sensor arrays and (B) the comparison of the absorbance strength between L-tyrosine (200 µM), aSyn monomers (50 µM) and aSyn fibrils (5 µM).

This result aligns with our expectations since standard FTIR measurements of amino acids typically require high concentrations (mg/mL) and long acquisition times (several hours) to produce clearly resolved peaks. In contrast, our SEIRA experiment of pure tyrosine under realistic concentrations and acquisition times detected only a faint signature, which is not even above the LoD level. Moreover, the absorbance intensity of 200 µM pure tyrosine only amounts to less than 3-6% of what is observed from pure aSyn fibril samples with only 3% of the molar concentration (5 µM) and from one-fourth of the molar concentration of pure aSyn monomers (50 µM) compared to that of the pure tyrosine (Figure S14 B).

We can thereby infer that the contribution from tyrosine side chains or from other amino acid side chains under our experimental conditions is negligible for the protein secondary structure analysis and therefore excluded from our analysis and methods, which are based on the established protocols. ^[50,73]^
